# Comparative Proteomics, Functional Characterization and Immunological Cross-Reactivity Studies on Russell’s Viper Venom from Two Distinct Geographical Regions in South India

**DOI:** 10.3390/ijms26199734

**Published:** 2025-10-07

**Authors:** Nisha Reghu, Sudharshan Rao, Dileepkumar Raveendran, Bipin Gopalakrishnan Nair, Muralidharan Vanuopadath

**Affiliations:** 1 School of Biotechnology, Amrita Vishwa Vidyapeetham, Kollam 690525, Kerala, India; 2 Indriyam Biologics Pvt. Ltd., SCTIMST-TIMED, 5th Floor, M S Valiathan Building, BMT Wing, Poojappura, Thiruvananthapuram 695012, Kerala, India

**Keywords:** snake venom, mass spectrometry, proteomics, Russell’s viper, antivenom, functional characterization, immunological cross-reactivity

## Abstract

Snakebite envenoming is a neglected tropical disease contributing to a significant number of morbidities and mortalities globally. Reports indicate that venom variation influences antivenom efficacy, which might affect treatment outcomes. The venom composition of *Daboia russelii* (Russell’s viper), one of the big four snakes in India, has been extensively studied from different geographical regions of India. Nonetheless, the Russell’s viper venom proteome from Kerala (Western Ghats region), together with its study in comparison with the same species’ venom from Tamil Nadu, has not been explored yet. In the current study, *Daboia russelii* venom from Irula (RVi) and the Western Ghats region in Kerala (RVwg) was characterized through mass spectrometry-based proteomics and few functional assays. The proteomics study identified 52 proteins from 14 snake protein families in RVi and 61 proteins from 17 snake venom protein families in RVwg. Some of the protein families, including DNase and hyaluronidase, as well as vascular endothelial growth factor, were exclusively identified in RVwg venom. Comparative functional analysis indicated that RVwg exhibited higher fibrinogenolytic and hyaluronidase activities, while RVi venom showed higher phospholipase A_2_ and L-amino acid oxidase activities. Through ELISA, RVi venom showed an end-point titration value of 1:24,300 for all the antivenoms used in this study, whereas for RVwg, compared to PSAV (Premium serums and vaccines) (1:2700), Virchow and VINS (both 1:8100) antivenoms showed better immunological cross-reactivity. Immunoblotting experiments indicated differential binding and recognition of antigenic epitopes present in both venoms by the polyvalent antivenoms used in the current study. All these findings highlight that the venom proteome varies according to the geographical location, and this significantly influences antivenom efficacy.

## 1. Introduction

Snakebite envenomation is a significant public health challenge that is overlooked, particularly in the tropics and subtropics. Worldwide, snakebites affect an estimated 5.4 million individuals annually, leading to 138,000 deaths [1,2]. Additionally, many envenomed victims suffer from permanent disabilities, including amputations and other impairments. A recent study showed that between 2000 and 2019, India accounted for about 1.2 million snakebite deaths, averaging approximately 64,000 fatalities annually [3]. This figure represents nearly half of the global mortality attributed to snakebites. The majority of snakebite incidents in India are caused by bites from the ‘big four’ snakes: Russell’s viper (*Daboia russelii*), Indian cobra (*Naja naja*), saw-scaled viper (*Echis carinatus*) and common krait (*Bungarus caeruleus*) [4]. Among these, Russell’s viper is responsible for the majority of mortalities and morbidities in India. The classical symptoms of Russell’s viper envenomation include coagulopathy; hemotoxicity; myotoxicity; nephrotoxicity; and, in some cases, neurotoxicity [5,6,7]. Epidemiological studies estimate that Russell’s viper (RV) accounts for approximately 43% of snakebite incidents in India, with some hospital-based reports attributing up to 80% of snakebite fatalities to this species [8,9]. Russell’s viper envenomation is a significant cause of mortality and morbidity in northern Kerala, with severe complications such as acute kidney injury coagulopathy and local tissue damage [6].

Snake venom is a complex combination of peptides and proteins, which account for about 90% of its dry weight [10]. Venom composition and potency may vary geographically, leading to differences in clinical manifestations and challenges in treatment strategies [11]. Apart from inter-species venom variation, numerous studies have also shown that intra-species snake venom variations are also not uncommon [12,13]. Multiple studies have reported considerable variation in the venom proteome profile of RV venom collected from the different parts of India, including the northern, western, eastern and southern locales (Table 1). These differences are reflected not only in the diversity of protein families but also in the abundance of key toxins. For instance, proteomics analyses have revealed that the venom of south Indian Russell’s viper predominantly contains neurotoxic phospholipase A_2_, contributing to 30% of the total proteome. But this activity dramatically declines while moving towards the western, northern and eastern regions of India [14]. Studies have also shown that geographical diversity in Russell’s viper venom has a major impact on its pharmacological and biochemical properties, including coagulant, hemorrhagic and neurotoxic effects [14,15]. These functional differences also have a direct impact on antivenom efficacy towards RV venom [11].

With antivenom being the only effective treatment option, geographical venom variations have proven to be quite problematic in controlling the severity of snakebites. Indian polyvalent antivenoms (PAVs) are typically raised against the venom of the big four snake species collected from Tamil Nadu. There are several reports that shows the inefficacy of these PAVs in neutralizing key venom components, notably PLA_2_ and other low-molecular-mass proteins that are critical contributors to the toxicity of viperid snake venom [22,23]. This limitation is often attributed to the poor immunogenicity of these low-molecular-weight proteins, resulting in inadequate antibody production during the immunization process [24]. Several studies have demonstrated the varied immunological cross-reactivity of polyvalent antivenom against Russell’s viper venom from different regions [17,22,24].

Even though RV venom from different parts of the country has been extensively studied, the composition of the RV venom proteome from Kerala (Western Ghats region) and its comparative analysis with RV venom from Tamil Nadu have not been explored in detail. In the current study, the RV venom proteomes from these two geographical locations and few functional studies are evaluated through in vitro approaches. The study also aims to assess the neutralization potential of different Indian polyvalent antivenoms using these venom samples.

## 2. Results

### 2.1. Proteomics Analysis of RVi and RVwg Venom

**Figure 1:**
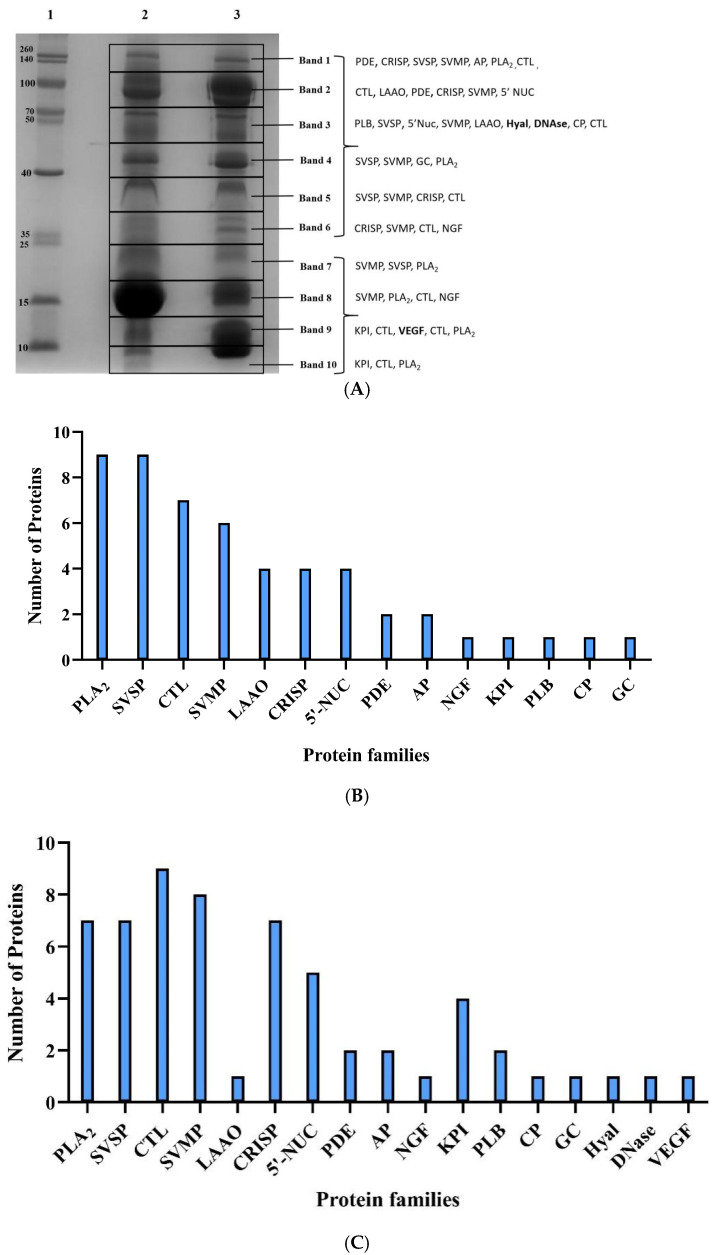
(**A**) 15% SDS-PAGE analysis of crude RVi and RVwg venom: Lane 1, molecular weight ladder; Lane 2, crude RVi venom resolved under reducing conditions; Lane 3, crude RVwg venom resolved under reducing conditions (PDE—phosphodiesterase; CRISP—cysteine-rich secretory protein; SVSP—snake venom serine protease; SVMP—snake venom metalloprotease; AP—aminopeptidase; PLA_2_—phospholipase A_2_; CTL—C-type lectin/snaclec; LAAO—L-amino acid oxidase; 5′-NUC—5′nucleotidase; PLB—phospholipase B; Hyal—hyaluronidase; CP—carboxypeptidase; GC—glutaminyl peptide cyclotransferase; NGF—nerve growth factor; KPI—Kunitz protease inhibitor; VEGF—venom endothelial growth factor). Letters highlighted in bold represent the protein family identified uniquely from RVwg. (**B**) Number of protein families identified from RVi venom. (**C**) Number of protein families identified from RVwg venom.

The profiling of crude RVi and RVwg venom revealed significant variation in the distribution of venom proteins. Distinct variations in banding patterns observed in the SDS-PAGE analysis indicate marked differences in the presence of different snake venom protein families present in both RVi and RVwg (Figure 1A). These proteins were identified through bottom-up proteomics approaches. After MS data acquisition, the obtained peptide information was compared against the Serpentes database.

Through our bottom-up proteomics analysis, 52 proteins belonging to 14 different snake protein families from RVi (Figure 1B) and 61 proteins belonging to 17 protein families from RVwg (Figure 1C) were identified. The proteins identified from RVi and RVwg after Liquid Chromatography–Mass Spectrometry/Mass Spectrometry (LC-MS/MS) analysis are shown in Supplementary Tables S1 and S2. Both venoms contain protein families representing phospholipase A_2_ (PLA_2_), snake venom serine protease (SVSP), snake venom metalloprotease (SVMP), C-type lectin (CTL), L-amino acid oxidase (LAAO), cysteine-rich secretory protein (CRISP), 5′ nucleotidase (5′ NUC), phosphodiesterase (PDE), aminopeptidase (AP), Kunitz protease inhibitors (KPI), phospholipase B (PLB) and nerve growth factor (NGF). Additionally, proteomic analysis revealed that certain protein families, including hyaluronidase (Hyal), vascular endothelial growth factors (VEGFs) and DNase, were exclusively identified in RVwg venom. Their corresponding peptide spectrum and fragmentation patterns are provided in Supplementary Table S3. As seen in Table 1, the DNase venom protein family was identified and reported for the first time in RV venom through our analysis. Interestingly, despite multiple reports characterizing the proteome of Russell’s viper venom from Tamil Nadu (venom collected from Irula) [18,19,20,21], our analysis uniquely identified the presence of aminopeptidase protein. However, three protein families previously reported in Irula venom—namely, hyaluronidase, VEGF and disintegrins [20,21]—were not detected in our study on RVi venom proteome.

### 2.2. RVwg Venom Exhibited Higher Fibrinogenolytic and Comparable Hyaluronidase Activities than RVi Venom

**Figure 2:**
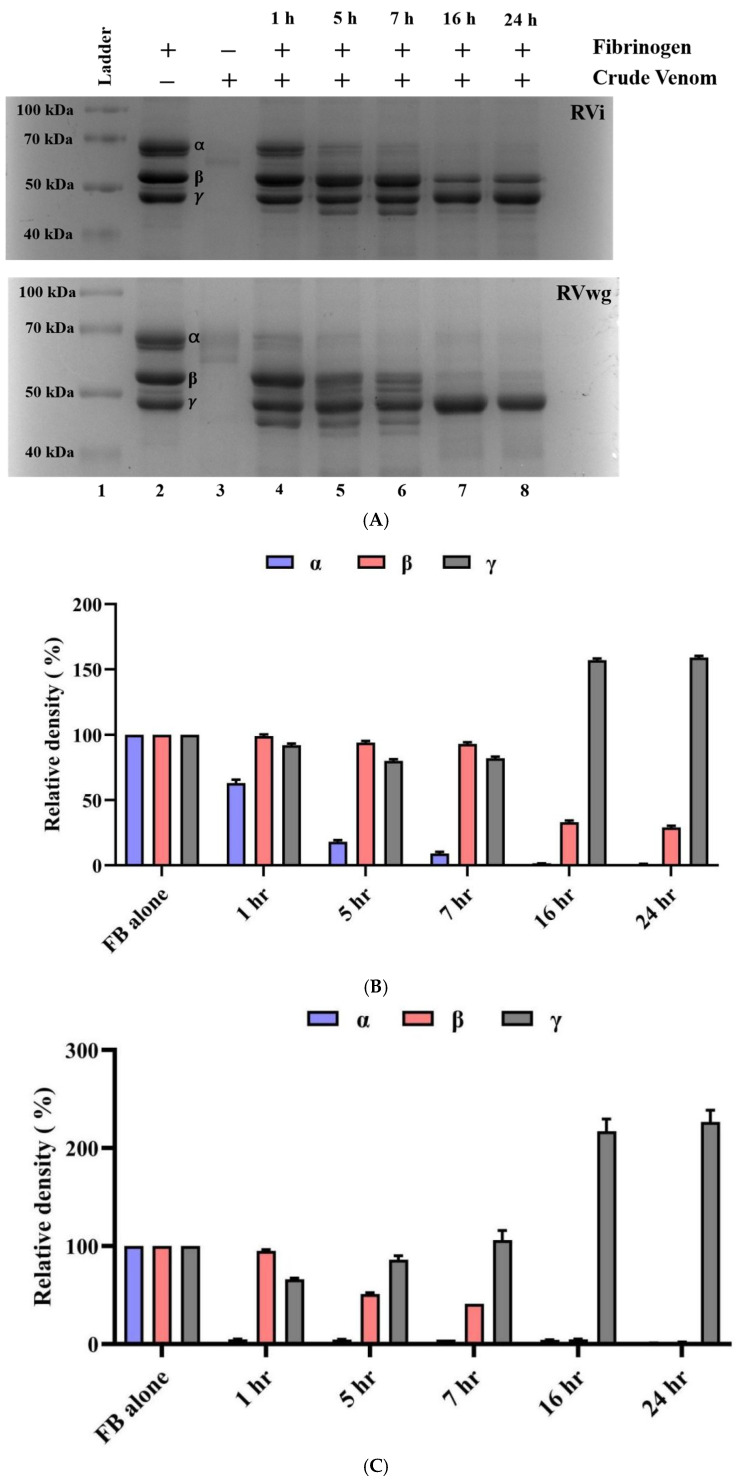
Fibrinogenolytic activity of crude RVi and RVwg venom. (**A**) Fibrinogenolytic activity of crude RVi and RVwg at different time points. Lane 1: molecular weight ladder; Lane 2: crude RVi and RVwg venom alone; Lane 3: fibrinogen alone; Lanes 4–8: crude RVi and RVwg incubated with fibrinogen at 1 h, 5 h, 7 h, 16 h and 24 h, respectively. (**B**) Densitometric analysis of fibrinogenolytic activity of RVi, showing the relative density of α, β and γ bands at different time points. (**C**) Densitometric analysis of fibrinogenolytic activity of RVwg, showing the relative density of α, β and γ bands at different time points.

To understand the impact of RVi and RVwg venom on fibrinogen degradation, they were evaluated for fibrinogenolytic activity, specifically targeting the Aα and Bβ subunits of human fibrinogen. Our findings indicate that fibrinogenolytic activity is increasing in RVi and RVwg in a time-dependent manner (Figure 2A–C). Notably, RVwg exhibited higher fibrinogenolytic activity, with the Aα subunit showing degradation within the first hour of incubation (Figure 2A,B), while Bβ-subunit degradation commenced by the fifth hour (Figure 2A,B). Surprisingly, RVi displayed a slower fibrinogen degradation pattern, where the Aα subunit started to degrade after one hour (Figure 2A,C) and the Bβ subunit exhibited only partial degradation after 16 h (Figure 2A,C).

**Figure 3:**
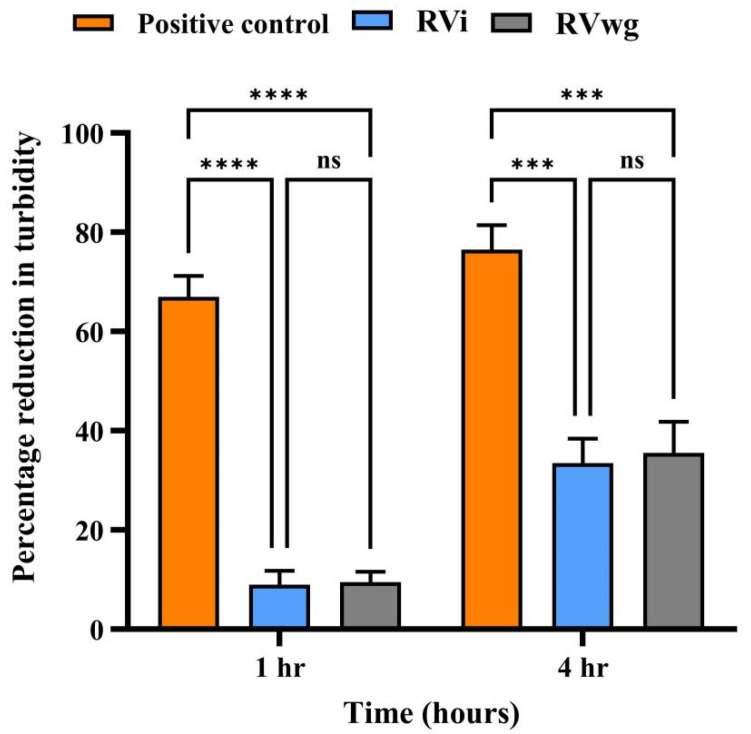
Hyaluronidase assay of crude RVi and RVwg venom. Percentage reduction in turbidity observed after 1 and 4 h of incubation using 1 μg of RVi and RVwg venom compared with the same amount of positive control (bovine testicular hyaluronidase). Each error bar represents the mean ± SD of triplicates. (*p* **** < 0.0001; *p* *** < 0.001; ns: non-significant).

To analyze the hyaluronidase activity of RVi and RVwg venom, we performed a turbidimetric assay in which the reduction in turbidity is indicative of the hyaluronidase activity at 400 nm. At a venom amount of 1 µg, RVwg demonstrated a 12% reduction in turbidity and RVi showed a 10% reduction in turbidity in the first hour, while RVwg showed a 38% reduction in turbidity and RVi showed a 35% reduction in turbidity in the fourth hour (Figure 3). These results indicate time- and dose-dependent increase in the hyaluronidase activity in both RVi and RVwg. Notably, RVwg venom consistently exhibited higher hyaluronidase activity, particularly at lower concentrations and early time points, indicating potentially higher levels of hyaluronidase activity in RVwg venom. This is also evident from our proteomics analysis, where peptides representing hyaluronidase were identified only in RVwg venom and not in RVi venom (Supplementary Tables S1 and S2). Though hyaluronidase activity was seen in RVi venom, peptides representing the same proteins were not identified through our proteomics analysis. This could be due to the methodology that we use for protein separation. For instance, in our study, we used only one-dimensional SDS-PAGE, followed by in-gel trypsin digestion and LC-MS/MS analysis. The identification could have improved if we had used orthogonal separation strategies for protein identification.

### 2.3. RVi Venom Exhibited Higher Phospholipase A_2_ and L-Amino Acid Oxidase Activities

**Figure 4:**
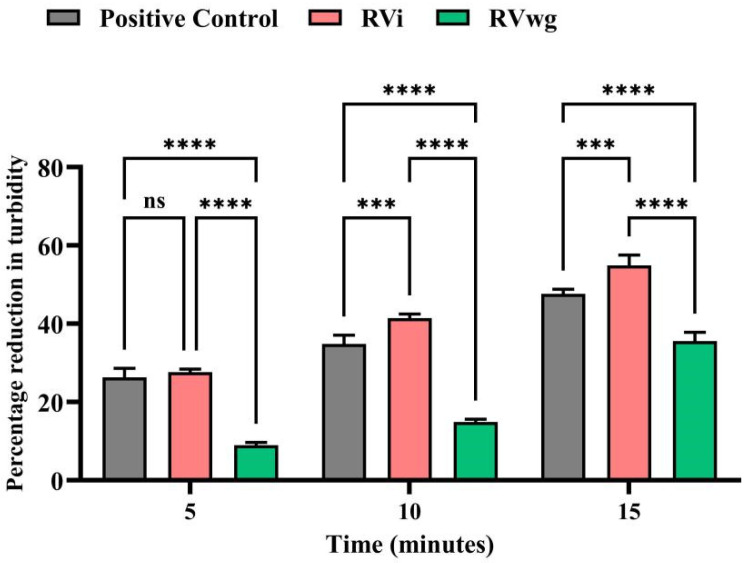
PLA_2_ assay of crude RVi and RVwg venom. Percentage reduction in turbidity was measured for a period of 15 min and compared with the positive control (purified PLA_2_ from bee venom). Each error bar represents the mean ± SD of triplicates. (*p* *** < 0.001; *p* **** < 0.0001; ns: non-significant).

**Figure 5:**
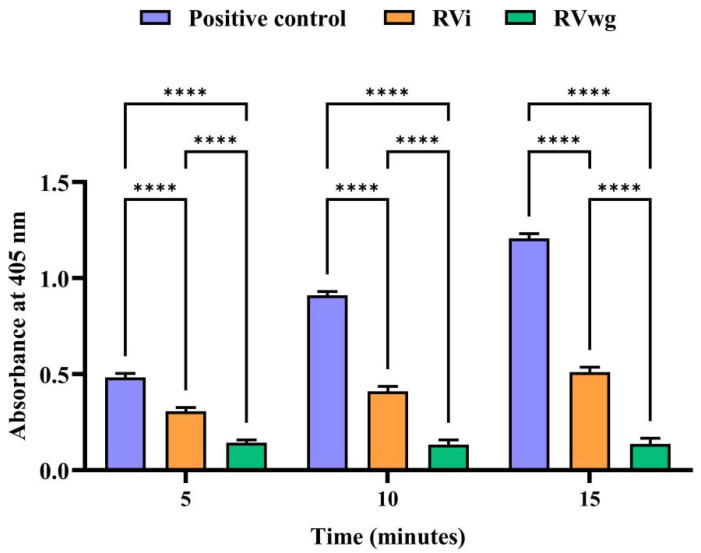
L-amino acid oxidase activity of crude RVi and RVwg venom. The change in absorbance at 405 nm, indicating H_2_O_2_ production by LAAO, was measured over a period of 15 min. Each error bar represents the mean ± SD of triplicates. (*p* **** < 0.0001).

Russell’s viper venom is generally known to exhibit higher PLA_2_ activity, and our findings reveal that RVi demonstrated greater PLA_2_ activity than RVwg (Figure 4). This observation corroborates our SDS-PAGE analysis results, where RVi demonstrated more intense protein bands, particularly in the low-molecular-mass regions around 12–15 kDa associated with PLA_2_ and other low-molecular-mass proteins (Figure 1A). From the results, it is also evident that the activity increases in a time-dependent manner. At all time points, a significant increase in PLA_2_ activity was observed in RVi venom when compared to RVwg. It is also noted that the PLA_2_ reading of RVi surpasses that of the positive control after the 5th minute, highlighting the increased PLA_2_ activity of RVi. Similarly, due to the significant influence of LAAO proteins inducing clinical manifestations post envenomation, we conducted LAAO assay to evaluate and compare the activity in RVi and RVwg venom. As seen in Figure 5, the LAAO activity was observed to be higher in RVi than in RVwg. A time-dependent increase in the LAAO activity was observed in RVi venom, whereas, even at the 15th minute, the LAAO activity seems to be negligible in RVwg venom. These results are in parallel with our LC-MS/MS analysis, where more peptides representing LAAO proteins were identified in RVi venom compared to RVwg venom (Figure 1B,C).

### 2.4. Immunological Cross-Reactivity of RVi and RVwg Venom Using Indian Polyvalent Antivenoms

**Figure 6:**
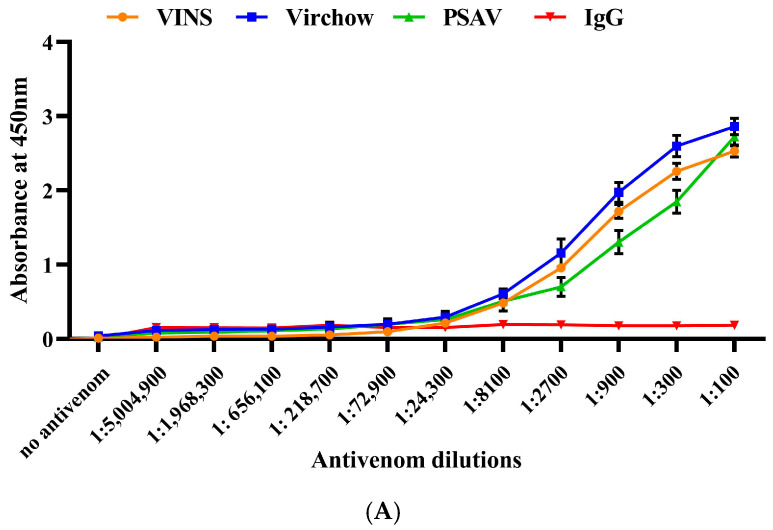

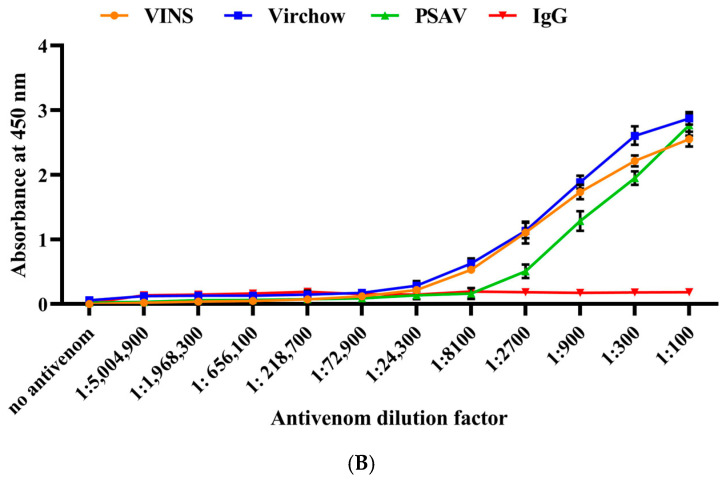
End-point titration ELISA of VINS, Virchow and PSAV antivenoms using crude (**A**) RVi and (**B**) RVwg venom. Different dilutions of antivenom were used to obtain the end-point titration values. Naive IgG from horse was used as the negative control. Each data point represents the mean ± SD of triplicates. (PSAV: Premium vaccines and serums).

The cross-reactivity of RVi and RVwg venom against commercial polyvalent antivenoms was evaluated using ELISA. As seen in Figure 6A,B, our findings reveal that all the antivenoms detected and bound to the antigenic epitopes present in both RVi and RVwg in a dose-dependent manner. Our data also reveal that all the antivenoms demonstrated improved immunoreactivity against RVi (Figure 6A). This is possibly because Russell’s viper venom (RVi) is included in the immunization mixture while generating antivenoms [4]. Consequently, the antibodies exhibit a high affinity for recognizing the specific epitopes found in RVi venom. All three of the antivenoms tested towards RVi venom showed better binding with the RVi venom at 1: 24,300 dilutions (Figure 6A). In contrast, RVwg showed a much lower binding potential than RVi, with VINS and Virchow antivenoms showing an end-point titration value of 1:8100, followed by PSAV at 1:2700 (Figure 6B).

**Figure 7:**
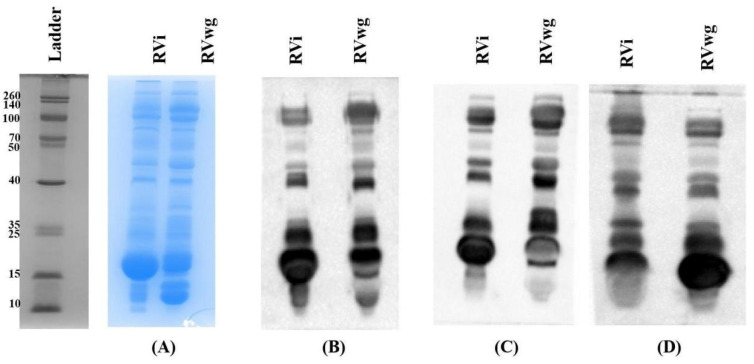
(**A**–**D**) Immunoblotting performed using RVi and RVwg towards different polyvalent antivenoms. (**A**) SDS-PAGE profile of crude RVi and RVwg. (**B**–**D**) Immunoblotting using (**B**) PSAV, (**C**) VINS and (**D**) Virchow.

The potential of the PAV in binding towards RVi and RVwg venom was evaluated through immunoblotting. For this, initially, 50 µg volumes of crude RVi and RVwg venom were separated through 15% SDS-PAGE (Figure 7A) and transferred to a PVDF membrane. As seen in Figure 7B–D, all the antivenoms detect and bind to the antigenic epitopes present in RVi venom effectively compared to RVwg. Previous reports on the venom of Russell’s viper and other snake species have suggested that most antivenoms fail to bind to proteins belonging to the low-molecular-mass region, especially below 20 kDa [20,22]. Interestingly, our results (Figure 7B–D) suggest that the antivenoms could bind to the RVi and RVwg proteins falling in this low-molecular-mass region.

## 3. Discussion

Snake venom comprises a diverse array of bioactive proteins and peptides that contribute to its overall toxicity and pharmacological effects. Therefore, understanding the venom composition is crucial for uncovering its pharmacological properties. Proteomic profiling uncovers the complexity of the venom composition by identifying both major and minor protein components through bottom-up proteomics [25]. This knowledge is essential for the development of targeted and effective antivenom therapies to address the clinical challenges associated with snakebite envenomation. Additionally, combining proteomic data with functional characterization allows researchers to better understand how venom proteins interact with various physiological systems in the victims [26]. Among the ‘big four’ snake species, *Daboia russelii* is the most studied in terms of assessing the venom variation across different geographical locations and assessing its functional characteristics. Table 1 clearly demonstrates that the composition of the venom proteome varies considerably based on geographical location. The identified differences in the protein families might also be due to the technology used for proteomics analysis. Even so, the variation in Russell’s viper venom collected from Irula has been demonstrated by different groups, which suggests that there is variation seen in the venom collected by the same vendor [15,16,18,19,20,21]. This might be attributed to the batch variation and the methodologies used for venom proteome identification. Notably, DNase was identified for the first time in Russell’s viper venom through our proteomic analysis.

Proteomic analysis of RVi and RVwg revealed the presence of diverse protein families. PLA_2_, one of the dominant proteins in RV venom, is known for its diverse pharmacological effects. It hydrolyzes glycerophospholipids (sn-2 position), resulting in the release of lysophospholipids and fatty acids [27]. Peptides representing nine and seven PLA_2_ proteins were identified from RVi and RVwg venom, respectively, through our analysis. As seen in Supplementary Tables S1 and S2, the identified peptides showed sequence similarity to the proteins reported from *Daboia* species (*Daboia russelii*, *Daboia siamensis* and *Daboia russelii pulchella*).

Numerous studies have reported that the abundance of PLA_2_ in RV venom varies across various locales [28,29]. Through our analysis, it was observed that both RVi and RVwg demonstrated significant levels of basic PLA_2_s which corroborates with other studies reporting higher numbers of basic PLA_2_s in RV venom from South India [15,19]. Moreover, the presence of neurotoxic PLA_2_ also varies according to geographical location. Southern Indian and Sri Lankan venoms have been found to contain significant proportions of neurotoxic PLA_2_s [18,19], correlating with the neurotoxic symptoms observed in envenomation cases from these areas.

Snake venom metalloproteases are class of enzymatic proteins that contribute to both local and systemic hemorrhage, in addition to interfering with the normal functioning of the blood coagulation cascade during Russell’s viper envenomation [30,31]. SVMPs are multi-domain, zinc-dependent enzymes that significantly contribute to hemorrhage and the degradation of fibrinogen [30]. Our proteomic analysis identified several proteins from the metalloprotease family, revealing eight SVMPs in the RVwg venom and six in the RVi venom. As detailed in Supplementary Tables S1 and S2, the SVMP derived from RVwg venom exhibited sequence similarity to peptides from two species of Daboia, as well as from other snake species like *Echis carinatus sochureki* and *Macrovipera lebetina*. The SVMP derived from RVi showed sequence similarity to peptides identified from *Ophiophagus hannah*, *Vermicella annulata*, *Daboia russelii russelii* and *Daboia siamensis.* The greater number of SVMPs in RVwg venom suggests increased fibrogenolytic activity, which is further supported by our functional experiments.

Snake venom serine proteases mainly target the hemostatic system. They interfere with the components of the fibrinolytic pathway coagulation cascade and kallikrein–kinin systems, leading to a disruption in hemostatic balance [31]. SVSPs are also one of the dominant proteins present in RVi; conversely, in RVwg, their prevalence is lower. Supplementary Table S2 shows the SVSPs identified from RVwg venom. Despite the observed differences in the total number of identified serine proteases, with RVi exhibiting a higher number, RVwg demonstrated a qualitative enrichment in fibrinogen-degrading enzymes. Specifically, the LC-MS/MS data identified two distinct β-fibrinogenase isoforms and one α fibrinogenase in RVwg, while RVi exhibited only a single isoform of each. α and β fibrinogenase contribute to fibrinogen depletion and the induction of coagulopathies, which can lead to hypofibrinogenemia and hemorrhagic effects [32,33].

C-type lectins, commonly referred to as Snaclecs, are non-enzymatic, calcium-dependent carbohydrate-binding proteins that fulfill a variety of biological functions, including the induction of coagulopathies. These proteins exhibit a range of roles, including the modulation of blood coagulation, platelet aggregation and immune responses [34]. The venom proteome profile of RVwg revealed the presence of nine C-type lectin isoenzymes, and all the derived peptides showed sequence homology to the peptides identified from several snake species, including *Daboia russelii limitis*, *Daboia siamensis*, *Vipera transcaucasiana* and *Macrovipera lebetina*. Meanwhile, the CTL peptides derived from RVi showed sequence similarity to the peptides derived from *Daboia siamensis*, *Pseudocerastes urarachnoides* and *Daboia russelii limitis*.

L-amino acid oxidases are homodimeric enzymes that contain flavin cofactors such as flavin adenine dinucleotide or flavin mononucleotide, which are covalently linked to the protein. The characteristic yellow coloration of venom with high LAAO content is attributed to the presence of riboflavin in these cofactors. LAAO exhibits diverse biological and pharmacological activities, including the induction of apoptosis, hemorrhage, cytotoxicity and platelet aggregation [35]. As seen in Supplementary Tables S1 and S2, in our LC-MS/MS analysis, one LAAO protein from RVwg and four LAAO proteins from RVi venom were identified. Our proteomic studies revealed the presence of proteins belonging to the CRISP family. They are single-chain polypeptides typically ranging between 20 and 30 kDa in molecular mass. These proteins exhibit highly conserved amino acid sequences with all 16 cysteine residues consistently preserved. CRISP plays a role in various physiological processes, particularly in modulating ion channels, which can influence muscle contraction and nerve signaling [36]. As seen in Supplementary Tables S1 and S2, our proteomic analysis identified a greater number of CRISP proteins in RVwg than in RVi.

Kunitz-type protease inhibitors possess a conserved motif comprising approximately 60 amino acids with three disulfide bonds [37]. Beyond their protease-inhibitory role, some KPIs specifically target voltage-gated potassium channels. These channels play critical roles in physiological functions such as fibrinolysis, host defense, coagulation and action potential transduction [37]. In our study, we identified seven different peptides representing four KPI proteins from RVwg, as well as one peptide representing one KPI protein from RVi. All the KPI peptides identified from both RVi and RVwg venom exhibited sequence similarity with peptides from the *Daboia* species.

DNases are endonuclease enzymes that hydrolyze the victim’s DNA, releasing adenosine, a nucleoside that enhances venom spread by increasing vascular permeability through vasodilation and/or by promoting platelet aggregation [38]. In our study, we identified one peptide from RVwg representing the DNAse family, which showed sequence similarity to the peptides derived from *Ophiophagus hannah*. Also, this study reports the identification of the DNase protein family through proteomics analysis in Indian Russell’s viper venom for the first time.

Venom hyaluronidase was first recognized in 1947 as a critical enzyme contributing to the ‘spreading effect’ of venom, achieved by degrading hyaluronic acid—a major component of the dermal extracellular matrix composed of glycosaminoglycans [39]. This enzymatic activity enhances the diffusion of fluids, including venom, within the skin. Additionally, it has been shown to contribute to increased local hemorrhage by promoting the dispersion of venom components [40]. Our proteomic analysis identified only one peptide representing the hyaluronidase enzyme from RVwg and showing sequence similarity to the peptides derived from *Echis ocellatus*. Peptides corresponding to hyaluronidase enzymes were not detected in RVi venom through our LCMS analysis, although a previous study reported their presence in Russell’s viper venom from Tamil Nadu [20]. This absence may also be attributed to limitations within the databases and the methodologies used, which failed to recognize certain peptides. While the database may not detect certain peptides, this does not imply that the corresponding proteins are absent from the venom.

5′ nucleotidase primarily functions by hydrolyzing nucleotides such as 5′-AMP, leading to the endogenous release of purines like adenosine. Adenosine is known to have multiple physiological effects, including immune modulation, platelet aggregation inhibition and vasodilation, all of which contribute to the systemic effects of envenomation [41]. In our study, five peptides representing 5′ NUC from RVwg and four from RVi were identified as showing sequence homology to different snake species. Snake venom phosphodiesterases are high-molecular-mass monomeric proteins that belong to the ectonucleotide family of metallo-enzymes. These phosphodiesterases cleave phosphodiester bonds in polynucleotides sequentially, releasing 5′ mononucleotides [42]. During envenomation, they can cause various pharmacological effects, including hypotension, edema, paralysis and inhibition of platelet aggregation [42]. Our proteomics study identified two phosphodiesterases from RVi and RVwg and one DNase enzyme from RVwg. As seen in Supplementary Tables S1 and S2, the peptides displayed sequence homology to proteins identified from *Macrovipera lebetina* and *Ophiophagus hannah*.

Phospholipase B is one of the proteins found in trace amounts in RV venom. These enzymes, also known as lysophospholipases, hydrolyze the ester bonds at both the sn-1 and sn-2 positions of glycerophospholipids within cell membrane [43]. During snakebite envenomation, snake venom phospholipases exhibit potent cytotoxic and hemolytic activities, leading to conditions such as myoglobinuria and cellular toxicity [43]. We identified two PLB proteins from RVwg and one from RVi. The enzyme glutaminyl-peptide cyclotransferase was also identified in the venom proteome of RVi and RVwg, as previously reported in other studies (Table 1) [19,44]. This enzyme plays a vital role in post-translational modifications, contributing to the structural stability of proteins, enhancing their resistance to aminopeptidase-mediated degradation, and facilitating protein–peptide interactions [45]. In addition to the protein families mentioned above, a few other minor protein families, including VEGFs, aminopeptidases and nerve growth factors, were determined through our proteomics analysis (Supplementary Tables S1 and S2). These proteins are reported to contribute to vascular permeability, protein degradation, and neuronal regeneration and modulation, thereby potentially influencing the overall effects of envenomation [46,47,48].

Information on the snake venom proteome is essential for linking the severity of clinical observations to venom composition variation. This is the first report on the proteomics of RV venom from Kerala (Western Ghats region). Additionally, understanding the intra-species variation is crucial for comprehending the differing clinical signs observed in snakebite cases. Since variation in the venom proteome can substantially influence the biochemical and pharmacological properties of snake venom, we conducted a panel of assays, including PLA_2_, hyaluronidase, LAAO and fibrinogenolytic assays. The PLA_2_ activity was found to be significantly higher in RVi compared to RVwg, which corroborates a previous study that showed PLA_2_ activity to be higher in RV venom from Tamil Nadu than Kerala [49]. This variation is well documented in RV venom, where the enzymatic activity shows considerable differences among populations from different regions of India [11]. Different isoenzymes of PLA_2_, categorized as either acidic or basic, are present in RV venom. Our proteomic analysis clearly demonstrated that both RVi and RVwg contain a robust mixture of these isoenzymes. This elevated activity of PLA_2_ in the venom proteome likely contributes to the development of hemorrhage, coagulopathy and edema, which correlate with clinical envenomation reports from South India [50,51]. Similarly, several studies substantiate the presence and activity of LAAOs in RV venom from different regions of India [11,19]. Although LAAOs are present in very low numbers in both RVi and RVwg venom, our functional analysis demonstrated higher LAAO activity in RVi compared to RVwg. Our enzymatic assay also demonstrated that RVwg exhibited comparatively higher hyaluronidase activity than RVi over different time points. This suggests that even though the enzyme was not detected in the proteomic analysis, its activity remains intact in crude venom. This sheds light on the importance of integrating functional assays with proteomic studies to fully capture the toxin diversity. The fibrinogenolytic activity of crude RVi and RVwg showed its tendency to cleave human fibrinogen consisting of Aα, Bβ and γ subunits. A previous report [11] showed that RV venom from Maharashtra, Tamil Nadu and Madhya Pradesh showed moderate fibrinogenolytic activity by cleaving the Aα subunit of fibrinogen, leaving the Bβ and γ subunits intact. Another study [52] showed that Russell’s viper venom from Tamil Nadu demonstrated an additional degradation of the gamma chain, while venom from Kerala primarily affected the Aα subunit. This contradicts our results, as we observed that the γ chain of fibrinogen was not affected in either of the venoms. Higher activity was found in RVwg, where the Aα subunit degraded within the first hour and the Bβ subunit degraded within 16 h. Conversely, in RVi, the Aα subunit started degrading at the 5th hour, with no effects on the Bβ and γ subunits. These observed variations, despite occurring in the same species, may be due to batch differences in the collected venom or differences in venom composition across geographic or individual samples, which may have implications for their differential effects on coagulation and envenomation pathology. While the present study did not investigate ecological factors, previous research has demonstrated that differences in habitat, prey availability and other ecological variables can also influence venom composition in snakes [49]. Such factors may also contribute to the geographic variation observed in Russell’s viper venom and warrant further investigation.

Although intravenous administration of equine-derived antivenom is the only mainstay treatment for snakebite envenoming, its effectiveness is limited by several significant factors. One notable issue is that low-molecular-mass toxins often trigger weak immune responses in the host, resulting in insufficient production of neutralizing antibodies against these highly toxic components [23]. Moreover, geographic and species-specific differences in venom composition can lead to antivenoms that are ineffective against toxins from populations not included in the immunization pool [22,53]. These challenges often require the use of large antivenom doses, which increase the possibility of adverse reactions [54]. Therefore, it is crucial to prioritize improvements in the potency and accessibility of antivenom formulations as part of global strategies for managing snakebites.

## 4. Materials and Methods

### 4.1. Reagents and Chemicals

3,3′,5,5′-Tetramethylbenzidine (TMB), Tween-20, sodium chloride (NaCl), dithiothreitol (DTT), ammonium bicarbonate, sodium phosphate, glycerol, formic acid, iodoacetamide (IAA), rabbit anti-horse secondary antibody (HRP-conjugated), hyaluronic acid, hyaluronidase, ammonium per sulphate, plasminogen-free fibrinogen, Peroxidase from horse radish (HRP), L-phenylalanine, 2,2′-azino-bis (3-ethylbenzothiazoline-6-sulfonic acid) (ABTS) and cetyltrimethylammonium bromide (CTAB) were obtained from Sigma-Aldrich (St. Louis, MO, USA). A BCA kit, prestained protein ladder, trypsin and purified PLA_2_ from bee venom were obtained from Thermo Fisher Scientific (Waltham, MA, USA). Tris, glycine, acrylamide, SDS, Commassie Brilliant Blue (CBB R-250) and TEMED were obtained from Bio-Rad Laboratories Inc (Hercules, CA, USA). Bovine Serum Albumin Fraction V and Bis-acrylamide were purchased from Himedia Laboratories Pvt Ltd., (Thane, Maharashtra, India). Furthermore, 96-well plates were obtained from Tarsons Products Ltd. (Kolkata, WestBengal, India). 96-well high-bind ELISA plates were obtained from Corning Inc., (Corning, NY, USA) and acetonitrile was obtained from Merck KGaA, (Darmstadt, Germany).

### 4.2. Venom and Antivenom

Venom from *Daboia russelii* was sourced from two locations with approval and permission from the respective state governments and forest departments: the Irula Snake Catchers Industrial Cooperative Society (license no-C.No.WL1/17197/2022-2(1) dated 20 December 2022) and the Western Ghats in Thiruvananthapuram (reference number 94/2009/F & WLD dated 25 February 2009). The venom supplied by Irula was a pooled sample, while the venom collected from the Western Ghats region was milked from two adult male specimens. RVwg venom from these two adult male specimens were collected in ice baths, and the samples were lyophilized immediately after venom collection. All the immunological cross-reactivity assays were performed using three Indian polyvalent antivenoms: VINS, PSAV and Virchow. All the venom and antivenom samples were stored at −80 °C until use. The antivenoms and RVwg venom were provided by Indriyam Biologics (Trivandrum, Kerala, India).

### 4.3. Protein Estimation

BCA assay was used to estimate the total protein concentrations in the venom samples using bovine serum albumin as the standard. First, 2 mg/mL of the BSA was serially diluted to prepare the working concentrations. Then, 10 µL from each BSA standard was mixed with BCA reagent (200 µL; prepared by mixing solutions A and B in a 1:50 ratio). The mixture was incubated at 37 °C for 30 min, and absorbance was measured at 562 nm using an Epoch 2 microplate spectrophotometer (BioTek Instruments, Inc., (Winooski, VT, USA).

### 4.4. SDS-PAGE and In-Gel Digestion

To resolve 100 µg of crude RVi and RVwg venom, 15% SDS-PAGE was used. The resolved proteins were stained using CBB R-250, and the image was visualized and captured using the Bio-Rad gel documentation system. After visualization, the protein bands were sliced into 20 bands, which were further cut into tiny pieces (0.5–1 mm^3^) and washed using a solution containing ammonium bicarbonate (25 mM) and acetonitrile (50%). The SDS-PAGE gel was excised into 20 individual bands, and each band was subjected to in-gel digestion followed by LC-MS/MS analysis. For ease of labeling and interpretation during data analysis, the results from the 20 bands were subsequently consolidated into 10 bands. Excess water in the gel pieces was removed by adding 100% acetonitrile to the gel pieces. The gel pieces were subjected to reduction (10 mM DTT; 45 min at 56 °C) and alkylation (55 mM Iodoacetamide; RT for 30 min in the dark) reactions. To remove the remaining iodoacetamide, the gel pieces were washed using ammonium bicarbonate (25 mM) and further treated using 100% acetonitrile until they appeared white in color. Furthermore, to these gel pieces, 25 μL of trypsin (13 ng/μL) prepared in 25 mM ammonium bicarbonate buffer (pH 8.0) was added. After incubation (overnight; 37 °C for 16 h), peptides were extracted. The pooled supernatant obtained from the extraction step was dried using a vacuum concentrator and was subjected to LC-MS/MS analysis as described below.

### 4.5. Tandem Mass Spectrometry

The LC-MS/MS analysis was carried out utilizing a nano HPLC system and a Q-TOF mass spectrometer (both from Agilent Technologies, Santa Clara, CA, USA), using a chip cube source as described previously [13]. The mobile phase A was water with 0.1% formic acid, and mobile phase B was a mixture of 90% acetonitrile and 10% water with 0.1% formic acid. A reversed-phase column from Agilent (ZORBAX 300 SB-C18, 75 μm × 150 mm, 5 μm) was used to separate the peptides. The capillary and nano pumps were set to deliver the mobile phases at a flow rate of 3 μL/min and 300 nL/min, respectively. The separation of the tryptic peptides was achieved using the following gradient: initial equilibration using 5% B for 2 min, 5–45% B for 30 min, 45–90% B for 45 min and 90% B for 2 min. MS and MS/MS data acquisition parameters were set with m/z ranges of 250–3000 and 50–3000, respectively, using nitrogen as the collision gas. The capillary voltage and fragmentor voltage were set at 2000 V and 150 V, respectively, with a dry gas flow rate of 4 L/min and a source temperature of 325 °C. The MS scan rate was configured at 4 spectra per second, while the MS/MS scan rate was set to 3 spectra per second. Up to five precursor ions were selected for fragmentation. For tryptic-digested samples, charge states of 2, 3 and greater than 3 were targeted. Active exclusion was applied after three spectra, with a releasing time of 0.5 min. Data acquisition was carried out using Agilent’s MassHunter software (Version 6.01).

### 4.6. Data Analysis by Mascot and Proteome Scaffold

The Mascot database (version 2.5.0, Matrix Science, London, UK) was used to analyze the MS data, and MassHunter Qualitative Analysis software (version 6.01) from Agilent was used to convert the raw data to Mascot generic format (.mgf) before performing the Mascot analysis. The following search parameters were used: database-Serpentes (Taxonomy ID: 8570), precursor ion mass tolerance; 10 ppm, peptide ion tolerance; 0.2 Da. Carbamidomethylation of cysteine was set as a fixed modification, and oxidation of methionine (M) and deamidation of asparagine (N) and glutamine (Q) were set as variable modifications. Trypsin was specified as the digestion enzyme, allowing for up to two missed cleavages. The decoy database option was enabled for false discovery rate assessment. Further validation of identified peptides and proteins was carried out using Proteome Scaffold software (version 4.5.0, Proteome Software Inc., Portland, OR, USA), with peptide and protein identification confidence thresholds set at 95% and 99%, respectively [55,56]. The data were filtered using the inbuilt peptide and Protein Prophet algorithms. Proteins identified by at least one unique peptide and not shared with other protein families were considered for the venom protein identification and classification process.

### 4.7. Functional Assays

#### 4.7.1. PLA_2_ Assay

The PLA_2_ activity was evaluated using a turbidimetric assay. The reaction substrate was freshly prepared by dissolving chicken egg yolk in a 0.9% NaCl solution. The assay was conducted in triplicate with a venom concentration of 1 μg prepared in 20 mM Tris-Cl buffer. Absorbance was recorded at 1 min intervals for 15 min at 740 nm. The PLA_2_ activity was determined based on the rate of turbidity decrease of the solution. Purified PLA_2_ from bee venom procured from Invitrogen (ThermoFisher, Waltham, MA, USA) was kept as the positive control.

#### 4.7.2. Hyaluronidase Assay

Hyaluronidase activity in RVi and RVwg crude venom was determined using a turbidimetric method. Venom samples were first pre-incubated at 37 °C for 10 min with a buffer composed of 0.2 M sodium acetate (pH 6.0) and 0.15 M NaCl. The enzymatic reaction was initiated by the addition of hyaluronic acid (0.015%) dissolved in the same buffer. The reaction mixtures were then incubated at 37 °C for 1 h and 4 h. To each well, 1 µg of crude venom was added and kept for incubation. After incubation, the reaction was stopped by adding 2.5% CTAB. The resulting precipitate was incubated for 8 min at room temperature, and the turbidity was measured at 400 nm. Control reactions were carried out using only substrate and buffer. Bovine testicular hyaluronidase enzyme was kept as a positive control.

#### 4.7.3. L-Amino Acid Oxidase Assay

A coupled enzymatic assay was used to estimate the LAAO activity in RVi and RVwg crude venom. A substrate mixture consisting of HRP, L-phenylalanine, ABTS and sodium phosphate buffer (0.1 M, pH 7.4) was added to the wells, followed by the addition of venom samples (RVi or RVwg) to initiate the reaction. Absorbance at 405 nm was recorded at 15 min intervals using a microplate reader.

#### 4.7.4. Fibrinogenolytic Assay

First, 15 µg of fibrinogen dissolved in phosphate-buffered saline (PBS) at pH 7.4 was mixed with known concentrations of venoms (1.5 µg) and incubated at 37 °C for different time points. After incubation, an equal volume of SDS gel-loading dye was added to the reaction mixture and heated at 95 °C for 5 min to stop the reaction. Subsequently, 15% SDS-PAGE gel was used to resolve the samples under reducing conditions, stained with CBB R-250, and the obtained bands were visualized and recorded using the Chemidoc MP Gel doc system from Bio-rad. The assay was performed thrice, and the densitometric analysis of the fibrinogen bands was computed using Image Lab software (version 5.2) from Bio-rad.

### 4.8. Immunological Cross-Reactivity Studies

#### 4.8.1. End-Point Titration ELISA

The ELISA plates were coated with 100 ng of venom and kept for incubation (4 °C overnight). After incubation, the wells were blocked with 2.5% BSA for 2 h to minimize non-specific interactions. Serial dilutions of antivenoms were prepared in 2.5% BSA at the following dilutions: 1:100, 1:300, 1:900, 1:2700, 1:8100, 1:24,300, 1:72,900, 1:218,700, 1:656,100, 1:1,968,300 and 1:5,004,900. After incubation (room temperature for 2 h), unbound antivenom was removed by washing with 0.1% PBST (Phosphate-Buffered Saline with Tween 20). Following this, secondary antibody (diluted 1:32,000 in 2.5% BSA) was added and kept for 2 h of incubation at room temperature. Following the washing steps, TMB/H_2_O_2_ substrate was added to the wells and incubated for 30 min at room temperature. The reaction was terminated by adding sulfuric acid (500 mM), and the absorbance was measured at 450 nm. Naïve horse IgG was used as the negative control. The experiments were performed in triplicate. The mean and SD of absorbance values obtained from three independent experiments were used to generate the graph.

#### 4.8.2. Immunoblotting

Venom proteins (50 μg of crude RVi and RVwg) were resolved under reducing conditions (100 V for 2 h) and subsequently transferred onto a PVDF membrane (wet transfer; 20 V for 18 h). Blocking was performed using 5% BSA to prevent any non-specific binding. The membrane was incubated overnight at 4 °C with the primary antibody (antivenoms from VINS, Virchow and PSAV) diluted 1:500 in the blocking buffer. Following incubation, unbound primary antibodies were removed by washing with 0.1% PBST. The membrane was then treated with an HRP-conjugated rabbit anti-horse secondary antibody (1:5000) for 3 h at room temperature, and the unbound secondary antibodies were removed by washing with 0.1% PBST. The blot was developed using an enhanced chemiluminescence solution, and the resulting signals were captured and documented using a gel-doc system.

### 4.9. Proteomics Data Availability

The mass spectrometry proteomics data were submitted to the ProteomeXchange Consortium [57] via the PRIDE partner repository [58] under dataset identifier PXD061802 (accession number).

### 4.10. Statistical Analysis

Statistical analysis was performed using GraphPad Prism (Graph pad software 10.5.0, San Diego, CA, USA). For PLA_2_, LAAO and hyaluronidase assays, three separate experiments were conducted, with three independent measurements recorded for each assay condition. The mean and SD of absorbance values obtained from the three experiments were used to plot the graph. Statistical analyses were performed using two-way ANOVA followed by Tukey’s multiple comparisons test to evaluate the difference between two groups. All results are represented as mean ± standard deviation (SD) of triplicate measurements unless stated otherwise.

## 5. Conclusions

This study investigated the comparative proteomics and functional characteristics of Russell’s viper venom from Kerala and Tamil Nadu. Consistent with previous research from our laboratory [13], our findings demonstrate variation in venom composition within the same species from different geographical regions. Although the two venoms exhibit a high degree of similarity in their protein composition and largely contain the same proteins, their functional activity differs, as demonstrated by our biochemical analysis. Functional assays corroborated the proteomics data, revealing distinct activity profiles. These results also underscore the necessity of optimized immunization strategies to produce region-specific antivenoms that can effectively address the clinical challenges of Russell’s viper envenomation in these distinct geographical areas. Numerous efforts are being made to strengthen the policies on mitigating snakebites and to streamline snakebite research in India, with a goal of reducing snakebite deaths by half by the year 2030 [59].

## Figures and Tables

**Table 1:** Comparison of protein families identified through proteomics analysis of Russell’s viper venom collected from different geographical locations in India. (+) indicates the presence of the venom protein family, and (-) indicates its absence. * indicates the current study.

Protein Family	Central India	Eastern India		Western India		Southern India					
	Madhya Pradesh [11]	Burdwan [16]	Nadia [16]	Mumbai [17]	Maharashtra [11]	Tamil Nadu [18]	Tamil Nadu [19]	Tamil Nadu [20]	Tamil Nadu [21]	Tamil Nadu (Current study) *	Kerala (Current Study) *
**PLA_2_**	+	+	+	+	+	+	+	+	+	+	+
**SVMP**	+	+	+	+	+	+	+	+	+	+	+
**SVSP**	+	+	+	+	+	+	+	+	+	+	+
**CRISPS**	+	+	+	+	+	+	+	+	+	+	+
**LAAO**	+	+	+	+	+	+	+	+	+	+	+
**CTLs**	+	+	+	+	+	-	-	+	+	+	+
**PLB**	-	-	+	+	-	-	+	-	-	+	+
**KPI**	+	+	+	+	+	+	+	+	+	+	+
**3FTx**	+	-	-	-	+	-	-	-	-	-	-
**NGF**	+	+	+	+	+	+	+	+	+	+	+
**VEGF**	+	+	+	+	+	+	+	+	+	-	+
**HYAL**	-	+	+	-	-	-	-	+	-	-	+
**PDE**	-	+	+	+	+	-	+	+	+	+	+
**5′NUC**	+	+	+	+	+	-	+	+	+	+	+
**Dis**	-	+	+	+	+	+	-	-	+	-	-
**AP**	-	+	-	-	-	-	-	-	-	+	+
**DNase**	-	-	-	-	-	-	-	-	-	-	+
**GC**	-	+	+	-	-	-	+	+	-	+	+
**CP**	-	-	-	-	-	-	+	-	-	+	+

## Data Availability

Data are contained within the article and Supplementary Materials.

## Supplementary Materials

The following supporting information can be downloaded at: https://www.mdpi.com/article/10.3390/ijms26199734/s1.
