# Supplementary material for: Comparative Proteomics, Functional Characterization and Immunological Cross-Reactivity Studies on Russell’s Viper Venom from Two Distinct Geographical Regions in South India
Source: Int J Mol Sci. 2025 Oct 7;26(19):9734. doi: 10.3390/ijms26199734 (PMC12524627; doi:10.3390/ijms26199734)
Supplement: Supplementary file 1 [file ijms-26-09734-s001.zip › Table S1.pdf]

Table S1 : Peptides representing various snake venom proteins in RVi identified through LC-MS. The individual mass spectrometric data obtained were analysed using mascot and further validated using Scaffold. The peptides are further grouped into different families of snake venom proteins. Amino acids marked in bold represent various modifications. M: oxidation of methionine (variable modification); N, Q: deamidation of asparagine and glutamine; c: carbamidomethylation of cysteine.

Sl.No.	Protein name	Accession number	Snake species	Molecular mass (kDa)	No.of unique peptides	MS/MS score	Sequence Coverage	Identified Peptides	Mascot Identity Score	Observed m/z (Z)	ppm	Band Number
**Phospholipase A2 (PLA2)**												
1	Basic phospholipase A2 VRV-PL-VIIIa	PA2B8_DABRR	*Daboia russelii*	14	20	736.9	96%	SLLEFGKMILEETGK	38.8	565.64(3)	2	9
								SLLEFGKMILEETGKLAIPSYSSYGcYcGWGGK	40.9	926.19(4)	3	9
								MILEETGKLAIPSYSSYGcYcGWGGK	39.5	976.45(3,4)	1	9
								**M**ILEETGKLAIPSYSSYGcYcGWGGKGTPK	40.4	832.39(3,4)	1	9
								LAIPSYSSYGcYcGWGGK	35.8	675.96(3)	1	10
								LAIPSYSSYGcYcGWGGKGTPK	38.8	803.70(3)	1	9
								LAIPSYSSYGcYcGWGGKGTPKDATDR	38.6	742.59(4)	<1	9
								DATDRccFVHDccYGNLPDcNPK	27.1	958.71(3)	2	9
								ccFVHDccYGNLPDcNPK	25	772.63(3)	3	9
								ccFVHDccYGNLPDcNPKSDR	27.7	669.27(4)	2	9
								V**N**GAIVcEK	38.3	495.75(2)	5	9
								GTScENRIcEcDKAAAIcFR	34.8	605.27(4)	<1	9

								IcEcDKAAAIcFR	36.4	538.58(3)	<1	9,4
								QNLNTYSK	37.6	484.25(2)	4	9
								QNLNTYSKK	37.9	365.86(3)	2	9
								KYMLYPDFLcK	40.1	739.36(2)	1	9
								KYMLYPDFLcKGELK	40.8	952.98(2,3)	2	7,9
								YmLYPDFLcK	38.5	675.32(2)	2	8,9
								YMLYPDFLcKGELK	40.5	888.94(2)	3	9
								YMLYPDFLcKGELKc	39.4	968.95(2)	2	9
2	Basic phospholipase A2 3	PA2B3_DABRR	*Daboia russelii*	14	8	306.9	92%	SLLEFGMMILEETGK	40.2	849.43(2,3)	3	4,9
								LAVPFYSSYGcYcGWGGK	35.6	691.31(2,3)	1	9
								VNGAIVcEQGTScENRIcEcDK	33.3	867.04(3)	2	9
								V**N**GAIVcEQGTScENR	36	897.89(2)	3	9
								NLNTYSKIYMLYPDFLcK	40.9	761.71(2,3)	2	1,4,9
								IYMLYPDFLcK	40.2	731.86(2)	2	9
								IYMLYPDFLcKGELK	40.7	945.48(2,3)	2	9
								IYMLYPDFLcKGELKc	40	684.00(3)	2	1,9
3	Phospholipase A2 1	PA21_DABRR	*Daboia russelii*	2	1	39.3	71%	NLFQFGEMILEK	39.3	734.88(2,3)	3	7,9
4	Chain A, Phospholipase A2 RV-7	pdb|1OQS|A	*Daboia siamensis*	14	2	73.5	29%	NLFQFGEMIL**Q**K	39.3	734.88(2)	3	7,9
								TATYSYSFENGDIVcGDNDLcLR	34.2	890.72(3,2)	2	9
5	Basic phospholipase A2	AAZ53183.1	*Daboia russelii*	15	1	31.5	13%	NPLSSYSNYGcYcGWGGK	31.5	690.62(3,2)	1	7,9

6	Basic phospholipase A2 RVV-VD	PA2B_DABRR	*Daboia russelii*	14	1	38.2	9%	NLFQFAEMIVK	38.2	670.35(2)	5	9
7	Phospholipase A2	ADG86232.1	*Vipera ursinii*	16	1	35.6	26%	IcEcDRAAAIcFR	35.6	821.36(2)	9	9
8	Chain B, Phospholipase A2	pdb|1OYF|B	*Daboia russelii pulchella*	14	1	39	90%	MILEETGRLAIPSYSSYGcYcGWGGK	39	985.75(3)	1	9
9	Neurotoxin acidic subunit alpha	AAB36097.1	*Pseudocerastes fieldi*	14	1	34.2	16%	EAVHSYAIYGcYcGWGGQGR	34.2	764.33(2,3)	<1	9
**Phospholipase B (PLB)**												
10	Phospholipase B-like 1	XP_026553321.1	*Pseudonaja textilis*	64	1	39.9	2%	FTAYAISGPPVEK	39.9	690.36(2)	4	3
**C-type Lectins (CTLs)**												
11	C-type lectin-like protein subunit 8	ABA86561.1	*Daboia siamensis*	18	4	157	51%	TWEDAEKFcTQQANGWHLASIESVEEANFVAQLASETLTK	39.6	1135.54(4)	1	9
								FcTQQANGWHLASIESVEEANFVAQLASETLTK	40.5	1227.26(3,4)	5	9
								QQcSSHWTDGSAVSYETVTK	36.7	757.67(3)	<1	9
								YHEWITLPcGDKNPFIcK	40.2	760.03(2,3,4)	<1	9
12	C-type lectin-like protein subunit 7	AAY63876.1	*Daboia siamensis*	18	3	112.6	19%	SSEEMDFVIR	36.7	606.78(2)	2	9
								FDFFWIGLR	38.2	600.81(2)	4	9
								WSDGVNLDYK	37.7	598.78(2)	<1	9

13	C-type lectin-like protein subunit 3	AAY63872.1	*Daboia siamensis*	17	3	109.6	20	VFTEE**M**NWADAEK	34	793.34(2)	2	9
								LDYKAWNEGTNcFVFK	40.1	664.65(2,3)	<1	9
								AWNEGTNcFVFK	35.5	736.83(2)	1	9
14	C-type lectin	UMW88214.1	*Pseudocerastes urarachnoides*	17	2	80.1	15%	TWEDAEKFcTEQVNGGHLVSFR	40.3	653.31(4)	8	9
								FcTEQVNGGHLVSFR	39.8	584.28(3)	<1	9
15	C-type lectin-like protein subunit 4	AAY63873.1	*Daboia siamensis*	17	2	78.1	18%	SMTcNFIAPVVcK	38.5	763.86(2)	2	7
								SMTcNFIAPVVcKF	39.6	837.40(2)	8	8
16	P31 alpha subunit	ADK22829.1	*Daboia russelii limitis*	18	1	39.5	8%	IIYVNWKEGESK	39.5	489.26(3)	<1	7
17	P68 alpha subunit	ADK22825.1	*Daboia siamensis*	18	3	114	27%	TPADYVWIGLR	40.5	645.84(2)	2	1,2,3,5,6,8,9
								WTDGSSVIYKNVIER	40	589.64(3)	<1	10
								TWFNLScGDDYPFVcK	33.5	1004.93(2,3)	3	10
**L-amino acid oxidase (LAAO)**												
18	secreted L-amino acid oxidase precursor	ACF70483.1	*Daboia russelii*	57	19	717.2	43%	EDDYEEFLEIAK	35.5	750.84(2)	3	3
								HIVIVGAG**M**SGLSAAYVLAGAGHK	38.3	765.75(3,4)	2	3
								EGWYANLGPMR	38.6	647.30(2)	3	3
								EGWYANLGPMRVPEK	40.2	873.93(2)	3	3
								LNEFVQETEnGWYFIK	40	672.99(3)	2	3
								KDPGLLKYPVKPSEAGK	35	366.21(3,4,	2	3

										5)		
								DPGLLKYPVKPSEAGK	37.6	425.49(4)	<1	3
								SAGQLYQESLGK	39.6	640.83(2)	4	3
								YDTYSTKEYLIK	39.7	762.38(2)	4	3
								RFDEIVGGMDQLPTSMYR	39.8	705.67(3)	2	3
								FDEIVGGMDQLPTSMYR	38.2	979.95(2,3)	3	3
								IQQNAEKVTVTYQTTQK	39.5	660.68(3)	5	3
								**n**LLLETADYVIVcTTSR	40.4	657.01(2,3)	6	3
								RITFKPPLPPK	29.2	431.94(3,4)	2	3
								ITFKPPLPPK	29.2	569.35(2,3)	5	3
								FWEDDGIQGGK6	36.9	626.28(2)	3	3
								KDLQTFcYPSIIQK	39.4	870.95(2,3)	4	3
								DLQTFcYPSIIQK	39.7	806.90(2)	3	3
								IFFAGEYTANAHGWIDSTIK	40.4	747.70(3)	3	3
19	L-amino-acid oxidase-like	XP_034277620.1	*Pantherophis guttatus*	57	1	39.9	3%	EAGH**q**VVILEAS**n**R	39.9	508.93(3)	3	2
20	L-amino oxidase	CAQ72894.1	*Echis ocellatus*	57	1	39.6	16%	SAGQLYQEALGK	39.6	632.83(2)	3	3
21	L-amino acid oxidase	ABN72538.1	*Ophiophagus hannah*	56	3	113.8	8%	EAGHEVVILEASDR	39.9	508.93(3)	3	2
								VTVFYEGLSTN**M**R	39.1	766.87(2)	3	2
								LVADYVLITATAR	34.8	469.27(3)	2	2
**Snake Venom Serine Proteinases (SVSPs)**												
22	Factor V	VSPA_DABS	*Daboia*	26	6	234.5	32%	FP**N**GLDKDIMLIR	39.2	511.61(3)	<1	5

	activator RVV-V alpha; SVSP	I	*siamensis*					RPVTYSTHIAPVSLPSR	37.2	627.68(3)	2	4
								ISTTEDTYPDVPHcTNIFIVK	40.8	817.40(3)	2	5
								WcEPLYPWVPADSR	38	888.41(2)	3	5
								TLcAGILK	39.1	438.25(2)	1	5
								TLcAGILKGGR	40.2	382.55(3)	3	5
23	Serine proteinase, homolog	CAB62591.1	*Macrovipera lebetina*	29	4	161	21%	FYcAGTLINQEWVLTAAR	40.3	705.02(3)	4	1,4
								NVPNEDQ**Q**IRVPK	39.5	513.27(3)	1	4
								WDKDIMLIR	40.7	397.22(3)	4	4,7
								TLcAGILQGGIDScK	40.5	531.60(2,3)	2	4
24	Serine protease VLSP-3 precursor	ADN04918.1	*Macrovipera lebetina*	28	1	40.3	9%	TSTHIAPLSLPSSPPSVGSVcR	40.3	750.72(3)	2	3
25	Snake venom serine proteinase 11	XP_015671557.1	*Protobothrops mucrosquamatus*	28	1	40.5	5%	T**N**KEWDKDI**M**LIR	40.5	420.47(4)	8	4
26	Serine endopeptidase	AUS82489.1	*Crotalus atrox*	28	1	40.4	4%	I**M**GWGTITPTK	40.4	610.82(2)	3	3,4,5
27	Snake venom serine protease VaSP1	VASP1_VIPAA	*Vipera ammodytes ammodytes*	22	1	41.2	32%	VIGGDEcNINEHPFLVALHTAR	41.2	616.31(4)	<1	4
28	Serine proteinase	AMB36345.1	*Vipera ammodyt*	29	1	40.4	6%	TLcAGILQGGIDTcK	40.4	536.27(3,2)	4	3,4

	SP-4		*es ammodytes*									
29	serine beta-fibrinogenase-like protein precursor	ADP88560.1	*Daboia siamensis*	28	1	39.7	6%	TSTYIAPLSLPSSPPR	39.7	562.97(2,3)	1	3
30	Serine alpha-fibrinogenase-like protein precursor	ADP88559.1	*Daboia siamensis*	28	1	40.4	4%	IMGWGSITSPK	40.4	588.81(2)	3	5
**Cysteine-rich secretory proteins (CRISPs)**												
31	cysteine-rich seceretory protein	ACE73567.1	*Daboia russelii*	27	3	103.9	17%	RPEIQNEIVDLHNSLR	39.3	484.01(3,4)	1	6
								MEWYPEAAANAER	35.8	769.34(2,3)	1	1,2,5,6
								cPAScFcHNEII	28.8	754.31(2)	<1	2,6
32	Cysteine-rich seceretory protein Ch-CRPKa, partial	ACE73560.1	*Crotalus horridus*	25	2	80.3	18%	SVDFDSESPRKPEIQNEIVDLHNSLR	40.6	756.88(4,5)	<1	6
								KPEIQNEIVDLHNSLR	39.7	477.01(4)	2	6
33	Snake venom CRISP precursor	KAG5858144.1	*Bothrops jararaca*	27	1	37.8	11%	WYPEAAANAER	37.8	639.30(2)	4	6

34	Cysteine-rich venom protein Bco13	CRVP_BOTCO	*Bothrops cotiara*	2	1	39.9	100%	SVDFDSESPRKPEIQ	39.9	578.61(3)	2	6
**5'-Nucleotidase (5'-NUC)**												
35	5'-nucleotidase	AHJ80886.1	*Macrovipera lebetina*	45	11	431.3	35%	ETPVLSNPGPYLEFR	40.3	859.94(2)	5	3
								IIALGHSGFFEDQR	40.5	795.40(2,3)	4	3
								QVPVVQAYAFGK	39.1	653.86(2,3)	4	2,3
								ASGNPILLNKDIPEDQVVK	37.3	684.04(3)	2	3
								DIPEDQVVKA**q**VNK	39.3	792.42(2)	2	2,3
								FHEcNLGNLIcDAVIYNNLR	40.4	812.39(3)	3	3
								HPDDNEWNHVSMcIVNGGGIR	36.8	803.03(3)	1	3
								HGQGTGELLQVSGIK	39.1	508.61(2,3)	2	3
								VVSLNVLcTK	37.6	566.82(2)	3	3
								cRVPTYVPLEMEK	40.8	811.40(2,3)	4	3
								VPTYVPLEMEK	40.1	653.34(2)	3	3
36	5'-nucleotidase, partial	BAN89427.1	*Ovophis okinavensis*	56	4	155.1	20%	HANFPILSANIRPK	35.6	526.63(3,4)	1	3
								YLGYLNVIFDDKGNVIK	39.2	657.69(3)	4	3
								GDSSNHSSG**n**LDISIVGDYIK	39.5	727.01(3)	1	3
								GDSSNHSSG**n**LDISIVGDYIKR	40.8	779.04(3)	2	3
37	Snake venom 5'-nucleotida	V5NTD_GLOBB	*Gloydius blomhoffii*	6	1	40.2	28%	SFELTILHTNDVHAR	40.2	584.97(2,3,4)	3	3

	se		*blomhoffii*									
38	5'-nucleotidase	XP_007427924.1	*Python bivittatus*	64	1	39.9	5%	VLLPSFLAAGGDGYHMLK	39.9	630.34(3)	2	3
**Nerve Growth Factor (NGF)**												
39	Nerve growth factor, NGF [Peptide, 117 aa]	AAA03282.1	*Vipera russelli, ssp. russelli*	13	2	75	21%	HWNSYcTTTDTFVR	34.7	596.60(3)	1	6,8
								INTAcVcVISR	40.3	646.83(2)	2	6,8
**Snake Venom Metalloproteinases (SVMPs)**												
40	Metalloproteinase	ABM87941.1	*Ophiophagus hannah*	69	3	104.8	6%	VFDMVNYITVVYK	40.6	795.91(2)	3	2
								NNLLHFSIWR	26.4	433.90(3)	<1	2
								ASYSEIEDIG**M**VDHR	37.8	579.93(3)	3	2
41	SVMP-Ver-17, partial	JAA74992.1	*Vermicella annulata*	57	1	37.6	4%	ISNEPHSEFSNcSVQEHQR	37.6	572.01(4)	8	1,2
42	Factor X activator heavy chain	ADJ67475.1	*Daboia russelii russelii*	70	2	70.8	0.04	NQcISLFGSR	38.8	591.29(2,3,12)	3	2
								DScFQENLKGSYYGYcR	32	714.65(3,2)	<1	2
43	Coagulation factor X activating enzyme light	AAB22478.1	*Daboia siamensis*	14	1	36.2	0.25	VLDcPSGWLSYEQHcYK	36.2		<1	3,4,5,6

	chain, RVV-X-light chain, LC1=metalloproteinase with disintegrin (platelet aggregation inhibitor)-like and C-type lectin-like domains											
44	Factor X activator light chain 2	ADJ67473.1	*Daboia russelii russelii*	18	1	39.5	0.06	FITHFWIGLR	39.5	430.58(3)	2	7
45	DSAIP, partial	AUF41660.1	*Daboia siamensis*	69	3	99.9	6%	VTLDLFGK	36	446.76(2)	3	3
								VTLDLFGKWK	36.6	402.90(3)	3	3
								AANGEcDVSDLcTGQSAEcPTDQFQR	27.3	1458.59(2)	5	3
**Kunitz Protease Inhibitor (KPI)**												
46	Kunitz protease inhibitor-IV	ABD24043.1	*Daboia russelii russelii*	9	1	37.3	14%	FcHLPVDSGIcR	37.3	487.56(3)	2	9,10
**Phosphodiesterase (PDE)**												

47	Phosphodiesterase, partial	AXL96599.1	*Borikenophis portoricensis*	101	1	40.7	2%	AERPDFLTLYIEEPDTTGHK	40.7	583.79(4)	5	1
48	phosphodiesterase	AHJ80885.1	*Macrovipera lebetina*	96	10	398.3	13%	AGYLETWDSLMPNINK	40.4	926.45(2)	4	1,2
								TLGMLMEGLK	40.6	546.79(2)	4	2
								TLGMLMEGLKQR	39.8	459.58(3)	6	1
								NVPKDFYTFDSEGIVR	39.8	629.65(3)	3	1
								DFYTFDSEGIVR	38	724.84(2)	1	1
								VNLMVDQQWMAVR	40.7	795.40(2)	4	1,2
								SMQAIFLAHGPGFK	40.4	501.93(3)	3	1
								cSSITDLGKVNER	40	493.58(3)	7	1
								SPPTSVPPSASDcLR	38.7	785.88(2)	3	1
								TFLPIFVNPVN	39.9	630.85(2)	7	1
**Aminopeptidases (APs)**												
49	xaa-Pro aminopeptidase 2 isoform X1	XP_039186984.1	*Crotalus tigris*	77	4	153.3	9	VEDYDQIGASLR	39.6	683.33(2)	7	1
								KLSLNEMYLLDSGGQYFDGTTDITR	41	946.46(3)	1	1
								VLMGNIDLSKLVFPPR	36	600.34(3)	4	1
								VVSLVPYAR	36.7	502.30(2)	7	1
50	xaa-Pro aminopeptidase 2	XP_015676063.1	*Protobothrops mucrosquamatus*	77	1	40.3	10%	LSWMTGFSGSEGTGVITLQK	40.3	1050.02(2)	4	1
**Carboxypeptidase (SCP)**												
51	Putative	AFJ49766.1	*Crotalus*	56	1	38.4	2%	QVGEFYQVIVR	38.4	669.36(2)	3	3

	serine carboxypeptidase CPVL-like		*adamanteus*									
**Glutaminyl-peptide Cyclotransferases (GC)**												
52	Glutaminyl-peptide cyclotransferases	AFE84762.1	*Daboia russelii*	42	3	116	13%	LQGLQAGWLVEEDTFQSHTPYGYR	40.6	932.45(3)	3	4
								LIFFDGEEAFVR	38.7	721.87(2)	4	4
								WSPSDSLYGSR	36.7	627.79(2)	<1	4
