# Supplementary material for: Comparative Proteomics, Functional Characterization and Immunological Cross-Reactivity Studies on Russell’s Viper Venom from Two Distinct Geographical Regions in South India
Source: Int J Mol Sci. 2025 Oct 7;26(19):9734. doi: 10.3390/ijms26199734 (PMC12524627; doi:10.3390/ijms26199734)
Supplement: Supplementary file 1 [file ijms-26-09734-s001.zip › Table S2.pdf]

**Supplementary Table S2**: Peptides representing various snake venom proteins in RVwg identified through LC-MS. The individual mass spectrometric data obtained were analysed using mascot and further validated using Scaffold. The peptides are further grouped into different families of snake venom proteins. Amino acids marked in bold represent various modifications. M: oxidation of methionine (variable modification); N, Q: deamidation of asparagine and glutamine; c: carbamidomethylation of cysteine.

| Sl. No. | Protein name | Accession number | Snake species | Molecular mass (kDa) | No.of unique peptides | MS/MS score | Sequence Coverage | Identified Peptides | Mascot Identity Score | Observed m/z (Z) | ppm | Band Number |
|---|---|---|---|---|---|---|---|---|---|---|---|---|
| **Phospholipase $A_2$ ($PLA_2$)** | | | | | | | | | | | | |
| 1 | Phospholipase A2-III, partial | AAP58959.1 | *Daboia siamensis* | 15 | 7 | 232.3 | 78% | EAVHSYAIYGcYcGWGGQGKPQDATDR | 35.7 | 762.34(4) | <1 | 9,10 |
| | | | | | | | | ccFVHDccYGTVNDcNPK | 25 | 769.29(2,3) | <1 | 10 |
| | | | | | | | | MATYSYSFENGDIVcGDNNLcLK | 35.3 | 891.05(2,3) | <1 | 10 |
| | | | | | | | | TVcEcDRAAAIcLGQNVNTYDK | 38.1 | 853.39(3) | <1 | 10 |
| | | | | | | | | AAAIcLGQNVNTYDK | 40 | 819.40(2,3) | <1 | 10 |
| | | | | | | | | AAAIcLGQNVNTYDKNYENYAISHcTEESEQc | 33.2 | 1251.53(3) | <1 | 10 |
| | | | | | | | | NYENYAISHcTEESEQc | 25 | 1067.41(2) | 1 | 10 |
| 2 | Basic phospholipase A2 | PA2B_DABRR | *Daboia ruselli* | 14 | 1 | 38.7 | 9% | NLFQFAE**M**IVK | 38.7 | 678.35(2,3) | 1 | 4,9,10 |
| 3 | Chain B, Phospholipase A2-II | pdb\|2H4C\|B | *Daboia siamensis* | 14 | 4 | 126.8 | 52% | YISYGcYcGWGGQGTPK | 34.2 | 977.42(2) | <1 | 10 |
| | | | | | | | | DATDRccFVHDccYAR | 25 | 702.61(3) | <1 | 10 |
| | | | | | | | | AVcEcDRVAAIcFR | 37.6 | 576.27(3) | 2 | 10 |
| | | | | | | | | YMLYSIFDcKEESDQc | 30 | 1044.42(2) | 2 | 10 |
| 4 | Basic phosphol | AAZ53178.1 | *Daboia siamensi* | 16 | 3 | 113.1 | 48% | AVcEcDRVAAIcLGQNVNTYNK | 39.4 | 852.40(3) | <1 | 10 |

| | | | | | | | | | | | | |
|---|---|---|---|---|---|---|---|---|---|---|---|---|
| | ipase A2 | | *s* | | | | | VAAIcLGQNVNTYNK | 40.9 | 555.62(2,3) | 1 | 10 |
| | | | | | | | | GYMFLSSYYcR | 32.8 | 723.81(2) | <1 | 10 |
| 5 | Acidic phospholipase A2 | AAZ53176.1 | *Daboia siamensis* | 16 | 2 | 66.1 | 53% | GKPQDATDRccFVHDccYGR | 31.8 | 501.21(5) | 3 | 10 |
| | | | | | | | | TATYSYSFENGDIVcGGDDPcLR | 34.3 | 866.37(2,3) | 4 | 10 |
| 6 | Basic phospholipase A2 | PA2B3_DABRR | *Daboia russelii* | 14 | 2 | 80.4 | 21% | SLLEFGMMILEETGK | 39.9 | 566.62(2,3) | 1 | 1,4 |
| | | | | | | | | IYMLYPDFLcK | 40.5 | 731.86(2) | 2 | 1,7,8 |
| 7 | Phospholipase A2 | AAA61467.1 | *Daboia russelii* | 4 | 2 | 75.4 | 67% | MILEETGKLAIPSYSSYGcYcGWGGK | 39.6 | 976.45(3) | 2 | 1 |
| | | | | | | | | LAIPSYSSYGcYcGWGGK | 35.8 | 1013.44(2) | <1 | 1 |
| **Phospholipase B (PLB)** | | | | | | | | | | | | |
| 8 | phospholipase B-like 1 | XP_026553321.1 | *Pseudonaja textilis* | 64 | 2 | 76.8 | 5% | QNSGTYNN**Q**YMILDTK | 37.4 | 945.93(2) | <1 | 3 |
| | | | | | | | | FTAYAISGPPVEK | 39.4 | 690.36(2) | 2 | 3 |
| 9 | Phospholipase B | QBF53421.1 | *Vipera ammodytes ammodytes* | 64 | 1 | 37 | 2% | YGLEFSYEMAPR | 37 | 731.84(2) | 1 | 3 |
| **L-amino acid oxidase (LAAO)** | | | | | | | | | | | | |
| 10 | Secreted L-amino acid oxidase precursor | ACF70483.1 | *Daboia russelii* | 57 | 24 | 924.5 | 47% | TSNPKHIVIVGAG**M**SGLSAAYVLAGAGHK | 38.9 | 706.37(4) | 7 | 2 |
| | | | | | | | | HIVIVGAGMSGLSAAYVLAGAGHK | 37.6 | 570.56(3,4) | <1 | 2 |
| | | | | | | | | HIVIVGAGMSGLSAAYVLAGAGHKVTVLEASERPGGR | 34.6 | 908.49(4,5) | <1 | 2 |
| | | | | | | | | NVKEGWYANLGPMR | 40.4 | 545.61(2,3) | 3 | 2 |
| | | | | | | | | EGWYANLGPMR | 38.3 | 647.31(2,3) | 1 | 2 |
| | | | | | | | | EGWYANLGPMRVPEK | 40.4 | 582.96(2,3) | <1 | 2 |

| | | | | | | | | | | | | |
|---|---|---|---|---|---|---|---|---|---|---|---|---|
| | | | | | | | | LNEFVQETENGWYFIK | 39.9 | 672.99(2,3) | 1 | 2 |
| | | | | | | | | KDPGLLKYPVKPSEAGK | 34.8 | 457.52(3,4,5) | 1 | 2 |
| | | | | | | | | DPGLLKYPVKPSEAGK | 37.4 | 566.98(2,3,4) | 2 | 2 |
| | | | | | | | | SAGQLYQESLGK | 39.4 | 640.83(2) | 1 | 2,3 |
| | | | | | | | | SAGQLYQESLGKAVEELKR | 39.5 | 527.28(3,4) | 5 | 2 |
| | | | | | | | | RFDEIVGGMDQLPTS**m**YR | 39.2 | 711(2,3) | <1 | 2,3 |
| | | | | | | | | FDEIVGGMDQLPTSMYR | 38.7 | 653.64(2,3) | <1 | 2 |
| | | | | | | | | AIEESVHFKAR | 39.6 | 429.57(3) | 1 | 2 |
| | | | | | | | | IQQNAEKVTVTYQTTQK | 39.3 | 660.68(3) | 1 | 2 |
| | | | | | | | | **N**LLLETADYVIVcTTSR | 40.1 | 657.01(2,3) | 8 | 2 |
| | | | | | | | | ITFKPPLPPK | 29.9 | 379.91(3) | <1 | 2 |
| | | | | | | | | KFWEDDGIQGGK | 39 | 690.33(2,3) | 2 | 2,3 |
| | | | | | | | | KFWEDDGIQGGKSTTDLPSR | 40.9 | 560.03(4) | 1 | 2 |
| | | | | | | | | FWEDDGIQGGK | 37.3 | 626.29(2) | 1 | 3 |
| | | | | | | | | FWEDDGIQGGKSTTDLPSR | 39.7 | 703.67(3) | <1 | 2 |
| | | | | | | | | KDLQTFcYPSIIQK | 39.5 | 870.95(2,3) | 1 | 2 |
| | | | | | | | | DLQTFcYPSIIQK | 39.6 | 806.90(2) | 2 | 2 |
| | | | | | | | | IFFAGEYTANAHGWIDSTIK | 40.5 | 747.70(3) | 1 | 2 |
| **Snake Venom Serine Proteinase (SVSP)** | | | | | | | | | | | | |
| 11 | Factor V activator RVV-V alpha SVSP | VSPA_DABSI | *Daboia siamensis* | 26 | 14 | 545.9 | 78% | VVGGDEcNINEHPFLVALYTSTSSTIHcGGALINR | 40.7 | 951.21(4) | <1 | 5 |
| | | | | | | | | EWVLTAAHcDR | 38.1 | 453.22(2,3) | <1 | 5 |
| | | | | | | | | NIRNEDEQIRVPR | 39 | 410.47(2,3,4) | 1 | 5 |

| | | | | | | | | | | | | |
|---|---|---|---|---|---|---|---|---|---|---|---|---|
| | | | | | | | | YFcLNTKFPNGLDKDIMLIR | 40.3 | 615.32(4) | <1 | 5 |
| | | | | | | | | FPNGLDKDIMLIR | 39.6 | 766.42(2,3) | 2 | 4,5 |
| | | | | | | | | LRRPVTYSTHIAPVSLPSR | 34.1 | 538.31(4) | 1 | 4 |
| | | | | | | | | RPVTYSTHIAPVSLPSR | 37.1 | 471.01(3,4,5) | <1 | 5 |
| | | | | | | | | ISTTEDTYPDVPHcTNIFIVK | 40.8 | 1225.60(2,3) | 1 | 5 |
| | | | | | | | | HKWcEPLYPWVPADSR | 39.8 | 1020.99(2,3,4) | 3 | 5 |
| | | | | | | | | WcEPLYPWVPADSR | 38.2 | 888.41(2,3) | 2 | 5 |
| | | | | | | | | TLcAGILK | 39.1 | 438.25(2) | 1 | 5 |
| | | | | | | | | TLcAGILKGGR | 39 | 382.55(3) | 5 | 5 |
| | | | | | | | | GGRDTcHGDSGGPLIcNGQIQGIVAGGSEPcGQHLKPAVYTK | 40 | 870.62(5) | <1 | 5 |
| | | | | | | | | DTcHGDSGGPLIcNGQIQGIVAGGSEPcGQHLKPAVYTK | 40.1 | 816.59(5) | <1 | 5 |
| 12 | Serine beta-fibrinogenase-like protein precursor | ADP88560.1 | *Daboia siamensis* | 28 | 1 | 39.8 | 6% | TSTYIAPLSLPSSPPR | 39.8 | 562.97(2,3) | 1 | 1,3 |
| 13 | Factor V activator RVV-V gamma - SVSP | VSPG_DABSI | | 29 | 2 | 76.3 | 58% | GGRDTcHGDSGGPLIc**N**GEMHGIVAGGSEPcGqHLKPAVYTK | 38.6 | 730.67(6) | 8 | 5 |
| | | | | | | | | DTcHGDSGGPLIcNGEMHGIVAGGSEPcGQHLKPAVYTK | 37.7 | 822.18(5) | 2 | 5 |
| 14 | Snake venom | VASP1_VIPAA | | 22 | 1 | 41.2 | 23% | VIGGDEcNINEHPFLVALHTAR | 41.2 | 616.31(3,4) | <1 | 4,7 |

| | | | | | | | | | | | | |
|---|---|---|---|---|---|---|---|---|---|---|---|---|
| | serine protease VaSP1 | | | | | | | | | | | |
| 15 | Serine alpha-fibrinogenase-like protein precursor | ADP88559.1 | *Daboia siamensis* | 28 | 1 | 40.4 | 4% | IMGWGSITSPK | 40.4 | 588.81(2) | 2 | 7 |
| 16 | serine beta-fibrinogenase precursor | ADN04917.1 | *Macrovipera lebetina* | 28 | 1 | 40.4 | 6% | TLcAGILQGGIDTcK | 40.4 | 803.90(2,3) | 2 | 2,4 |
| 17 | Serine protease VLSP-1 precursor | ADN04916.1 | *Macrovipera lebetina* | 29 | 1 | 40.1 | 5% | SYTPWDKDIMLIR | 40.1 | 546.62(3) | 1 | 4 |
| **Snake Venom Metalloproteinase (SVMP)** | | | | | | | | | | | | |
| 18 | hemorrhagic metalloproteinase | AAZ39880.1 | *Daboia russelii* | 70 | 10 | 382.6 | 14 | ASLLVATSER | 38.5 | 523.80(2) | <1 | 2 |
| | | | | | | | | VPLVGLEIWK | 30.4 | 577.35(2) | 2 | 1,2,3,4,5,6 |
| | | | | | | | | VPLVGLEIWKNR | 32.5 | 475.29(3) | 2 | 2 |
| | | | | | | | | RFLTEHNPEcIINPPLR | 40.5 | 702.70(3,4) | <1 | 2 |
| | | | | | | | | FLTEHNPEcIINPPLR | 41.1 | 650.67(2,3,4) | <1 | 1,2,3,4 |
| | | | | | | | | FLTEHNPEcIINPPLRTDIVSPPAcGNELLER | 40.3 | 926.22(4) | 2 | 2 |
| | | | | | | | | TDIVSPPAcGNELLER | 39.8 | 590.96(2,3) | 7 | 1,2,3 |
| | | | | | | | | SPGNQIPcLPYYIPSDENK | 39.7 | 1096.52(2,3) | 2 | 1,2,5,6 |
| | | | | | | | | LYcSYKSPGNQIPcLPYYIPSDENKGMVDHGTK | 39.8 | 767.16(5) | <1 | 2 |

| | | | | | | | | | | | | |
|---|---|---|---|---|---|---|---|---|---|---|---|---|
| | | | | | | | | SPGNQIPcLPYYIPSDENKG**M**VDHGTK | 40 | 759.11(3,4,5) | 1 | 1,2 |
| 19 | RVV-X heavy chain | AUF41652.1 | *Daboia siamensis* | 69 | 8 | 287.86 | 13% | KSHDNALLFTDMR | 40.3 | 516.59(3) | <1 | 2 |
| | | | | | | | | SHDNALLFTDMR | 39.3 | 710.34(2,3) | 2 | 1,2,3 |
| | | | | | | | | cILYPPLR | 39.26 | 516.29(2) | <1 | 1 |
| | | | | | | | | LKPGAEcG**N**GLccYQcK | 33.6 | 672.62(3) | 3 | 2 |
| | | | | | | | | GYcY**N**GDcPIMR | 26.6 | 753.79(2,3) | 2 | 1,2,3,6,7 |
| | | | | | | | | NQcISLFGSR | 39 | 591.29(2) | <1 | 1,2,4 |
| | | | | | | | | ANVAKDScFQENLKGSYYGYcR | 37.8 | 658.30(3,4) | <1 | 2 |
| | | | | | | | | DScFQENLKGSYYGYcR | 32 | 716.30(3) | <1 | 2 |
| 20 | DSAIP, partial | AUF41660.1 | *Daboia siamensis* | 69 | 10 | 353.7 | 18% | ASLLNLTPEQR | 36.6 | 621.35(2) | 1 | 2,3 |
| | | | | | | | | INVLPEAK | 31.7 | 442.27(2) | 2 | 3 |
| | | | | | | | | VTLDLFGK | 35.7 | 446.76(2) | 1 | 1,2,3,6 |
| | | | | | | | | VTLDLFGKWK | 36.9 | 402.90(2,3) | <1 | 3 |
| | | | | | | | | WKASDLLNHR | 37.7 | 413.89(3) | <1 | 3 |
| | | | | | | | | ScIMSGTLScEASNR | 32.7 | 836.86(2,3) | <1 | 2,3,6 |
| | | | | | | | | MPQcILNKPLK | 37.8 | 447.92(3) | <1 | 3 |
| | | | | | | | | AA**N**GEcDVSDLcTGQSAEcPTDQFQR | 27.4 | 973.06(2,3) | <1 | 3 |
| | | | | | | | | QcISLFGSR | 38.7 | 534.27(2) | 2 | 1,2,3,6,7 |
| | | | | | | | | LYcFDNLPEHK | 38.5 | 479.23(3) | 2 | 3 |
| 21 | Metalloproteinase, partial | ADI47595.1 | *Echis carinatus sochureki* | 30 | 1 | 39.8 | 6% | TDIVSPPVcGNDLLER | 39.8 | 595.63(2,3) | <1 | 1,2,3 |
| 22 | coagulation factor X activating enzyme | AAB22478.1 | *Daboia siamensis* | 14 | 1 | 35.9 | 38% | VLDcPSGWLSYEQHcYK | 35.9 | 714.65(3) | 1 | 8 |

| | | | | | | | | | | | | |
|---|---|---|---|---|---|---|---|---|---|---|---|---|
| | light chain | | | | | | | | | | | |
| 23 | Factor X activator heavy chain precursor | AAQ17467.1 | *Macrovipera lebetina* | 69 | 1 | 40.8 | 5% | TAVIMAHELGHNLGMYHDR | 40.8 | 433.81(4,5) | 2 | 2 |
| 24 | factor X activator light chain 2 | AAW69869.1 | *Daboia russelii* | 18 | 1 | 37.5 | 7% | cFVLEKESGYR | 37.5 | 463.23(3) | <1 | 7,8 |
| 25 | Factor X activator light chain 2 | ADJ67473.1 | *Daboia russelii russelii* | 18 | 2 | 77.2 | 13% | FITHFWIGLR | 39.1 | 430.58(3) | 3 | 7 |
| | | | | | | | | cFVL**Q**KESGYR | 38.1 | 463.23(3) | <1 | 7,8 |
| 26 | PREDICTED: zinc finger protein 501-like | XP_013916710.1 | *Thamnophis sirtalis* | 51 | 1 | 34.1 | 2% | RTTLSPVFR | 34.1 | 359.55(3) | <1 | 5 |
| **Cysteine-rich secretory protein (CRISP)** | | | | | | | | | | | | |
| 27 | Cysteine-rich seceretory protein Dr-CRPK | ACE73567.1 | *Daboia russelii* | 27 | 7 | 262.2 | 28% | RPEIQNEIVDLHNSLR | 40.2 | 645.01(3,4) | 2 | 6 |
| | | | | | | | | RPEIQNEIVDLHNSLRR | 38.3 | 523.04(4) | <1 | 6 |
| | | | | | | | | SVTPTASNMLK | 40.4 | 574.80(2) | 2 | 6 |
| | | | | | | | | SVTPTASNMLK**M**EWYPEAAANAER | 39.6 | 895.09(3) | 1 | 6 |
| | | | | | | | | MEWYPEAAANAER | 35.2 | 769.34(2,3) | <1 | 1,2,5,6 |
| | | | | | | | | cGENIYMSPYPMK | 32.9 | 795.34(2) | 2 | 5,6 |
| | | | | | | | | TKcPAScFcHNEII | 35.6 | 868.88(2) | 2 | 6 |
| 28 | Cysteine-rich seceretor | ACE73560.1 | *Crotalus horridus* | 25 | 2 | 76 | 18% | SVDFDSESPR | 35.4 | 569.75(2) | 3 | 6 |
| | | | | | | | | SVDFDSESPRKPEIQNEIVDLHNSLR | 40.6 | 605.71(5) | <1 | 6 |

| | | | | | | | | | | | | |
|---|---|---|---|---|---|---|---|---|---|---|---|---|
| | y protein Ch-CRPKa, partial | | | | | | | | | | | |
| 29 | Cysteine rich secretory protein, partial | ASU45033.1 | *Daboia russelii* | 29 | 1 | 39.5 | 16% | KPEIQNEIVDLHNSLR | 39.5 | 635.68(3,4) | 2 | 6 |
| 30 | Cysteine-rich venom protein | CRVP_ECHCO\ | | 25 | 1 | 40 | 11% | SVNPTASN**M**LR**M**EWYPEAAANAER | 40 | 685.82(4) | 9 | 6 |
| 31 | Snake venom CRISP precursor | KAG5858144.1 | *Bothrops jararaca* | 27 | 1 | 37.9 | 11% | WYPEAAANAER | 37.9 | 639.30(2) | 1 | 6 |
| 32 | Piscivorin | AAO62994.1 | *Agkistrodon piscivorus piscivorus* | 27 | 1 | 39.4 | 12% | KPEI**Q**NQIVDLHNSLR | 39.4 | 477.01(3,4) | 1 | 6 |
| 33 | Ophanin | AAO62996.1 | *Ophiophagus hannah* | 27 | 1 | 37.6 | 4% | EIVDLHNSLR | 37.6 | 598.32(2,3) | 1 | 6 |
| **C-type Lectin (CTL)** | | | | | | | | | | | | |
| 34 | P68 alpha subunit | ADK22827.1 | *Daboia russelii limitis* | 18 | 5 | 188.9 | 33% | TPADYVWIGLR | 40.3 | 645.85(2) | 1 | 1,2,3,5,6,8,9 |
| | | | | | | | | WTDGSSVIYK | 37.6 | 578.29(2) | 2 | 8 |
| | | | | | | | | WTDGSSVIYKNVIER | 40.1 | 883.96(2,3) | <1 | 8 |
| | | | | | | | | FIKNcFGLEK | 37.5 | 419.22(3) | 1 | 8 |
| | | | | | | | | TWFNLScGDDYPFVcK | 33.4 | 1004.93(2) | 2 | 8 |
| 35 | C-type lectin-like | AAY63876.1 | *Daboia siamensi* | 18 | 2 | 75.1 | 13% | SSEEMDFVIR | 37.4 | 606.78(2,3) | <1 | 2,6,8,9,10 |
| | | | | | | | | WSDGVNLDYK | 37.7 | 598.78(2) | <1 | 8,10 |

| | | | | | | | | | | | | |
|---|---|---|---|---|---|---|---|---|---|---|---|---|
| | protein subunit 7 | | *s* | | | | | | | | | |
| 36 | C-type lectin-like protein subunit 4 | AAY63873.1 | *Daboia siamensis* | 17 | 4 | 151.8 | 29% | VFTEE**M**NWADAEK | 34.3 | 793.35(2) | <1 | 5,10 |
| | | | | | | | | ALAEESYcLIMITHEK | 40.4 | 636.65(3) | 2 | 8 |
| | | | | | | | | SMTcNFIAPVVcK | 38.4 | 763.86(2) | <1 | 8,9 |
| | | | | | | | | SMTcNFIAPVVcKF | 38.7 | 837.40(2) | <1 | 8 |
| 37 | C-type lectin | UMW88214.1 | *Pseudocerastes urarachnoides* | 17 | 3 | 117.9 | 23% | KTWEDAEKFcTEQVNGGHLVSFR | 40.8 | 548.47(4,5) | 2 | 10 |
| | | | | | | | | FcTEQVNGGHLVSFR | 40.6 | 584.29(3) | 7 | 8,10 |
| | | | | | | | | AWSGEPNcYVAK | 36.5 | 691.31(2) | 1 | 8,10 |
| 38 | c-type lectin | ABW82659.1 | *Macrovipera lebetina* | 18 | 1 | 36.5 | 6% | HLATIEWLGK | 36.5 | 584.33(2,3) | 3 | 10 |
| 39 | C-type lectin-like protein subunit 3 | AAY63872.1 | *Daboia siamensis* | 17 | 1 | 35.5 | 17% | AWNEGTNcFVFK | 35.5 | 736.84(2) | <1 | 10 |
| 40 | C-type lectin-like protein subunit 8 | ABA86561.1 | *Daboia siamensis* | 18 | 1 | 39.8 | 6% | SKYHAWIGLR | 39.8 | 615.84(2) | <1 | 10 |
| 41 | C-type lectin | UMW88257.1 | *Vipera transcaucasiana* | 18 | 2 | 70.1 | 14% | GFSWEWTDGSSTK | 35 | 744.32(2) | 2 | 10 |
| | | | | | | | | NAFLcQcRF | 35.1 | 608.27(2) | <1 | 10 |
| 42 | C-type lectin J | JAC96617.1 | *Echis coloratus* | 18 | 1 | 39.4 | 8% | SSADYVWIGLWNK | 39.4 | 769.88(2) | 2 | 6 |
| **Phosphodiesterase (PDE)** | | | | | | | | | | | | |
| 43 | Phosphodiesterase | AHJ80885.1 | *Macrovipera lebetina* | 96 | 13 | 512.4 | 20% | AGYLETWDSLMPNINK | 40.7 | 926.45(2,3) | 4 | 1 |
| | | | | | | | | AATYFWPGSEVK | 38.8 | 678.33(2) | <1 | 1 |
| | | | | | | | | FGPVSGEII**M**ALQ**M**ADR | 40.1 | 622.98(3) | 1 | 1 |
| | | | | | | | | TLG**M**LMEGLK | 39.1 | 554.79(2,3) | <1 | 1 |
| | | | | | | | | NVPKDFYTFDSEGIVR | 40.2 | 943.97(2,3) | <1 | 1 |

| | | | | | | | | | | | | |
|---|---|---|---|---|---|---|---|---|---|---|---|---|
| | | | | | | | | DFYTFDSEGIVR | 38.6 | 724.84(2) | 4 | 1 |
| | | | | | | | | IDKVNL**M**VDQQWMAVR | 40.9 | 654.67(3) | 2 | 1 |
| | | | | | | | | VNLMVDQQWMAVR | 40.6 | 795.40(2) | <1 | 1 |
| | | | | | | | | SMQAIFLAHGPGFK | 40.4 | 501.93(2,3) | 1 | 1 |
| | | | | | | | | SPPTSVPPSASDcLR | 39 | 785.88(2,3) | 1 | 1 |
| | | | | | | | | VLSFILPHRPDNSESc ADTSPDNLWVEER | 40.5 | 846.66(4) | 1 | 1 |
| | | | | | | | | QPLPETLQLK | 34.4 | 583.34(2) | <1 | 1,2 |
| | | | | | | | | TFLPIFVNPVN | 39.1 | 630.85(2) | <1 | 1 |
| 44 | phosphodiesterase, partial | AXL96599.1 | *Borikenophis portoricensis* | 101 | 1 | 41 | 4% | AERPDFLTLYIEEPDT TGHK | 41 | 583.79(3,4) | 1 | 1 |
| **5'-Nucleotidase (5'-NUC)** | | | | | | | | | | | | |
| 45 | 5'-nucleotidase, partial | AHJ80886.1 | *Macrovipera lebetina* | 45 | 10 | 386.9 | 26% | LTTLGVNKIIALGHSGF FEDQR | 37.4 | 604.83(4) | 3 | 2 |
| | | | | | | | | IIALGHSGFFEDQR | 40.7 | 530.61(2,3) | <1 | 2 |
| | | | | | | | | QVPVVQAYAFGK | 38.1 | 653.86(2) | 2 | 2,3 |
| | | | | | | | | ASGNPILLNKDIPEDQ VVK | 37.1 | 684.04(2,3) | <1 | 2,3 |
| | | | | | | | | HGQGTGELLQVSGIK | 38.9 | 508.61(2,3) | <1 | 2 |
| | | | | | | | | HGQGTGELLQVSGIK VVYDLSQKPGSR | 37.7 | 714.14(4) | 2 | 2 |
| | | | | | | | | VVSLNVLcTK | 37.4 | 566.82(2) | 1 | 2 |
| | | | | | | | | cRVPTYVPLEMEK | 40.9 | 541.27(3) | <1 | 2 |
| | | | | | | | | VPTYVPLEMEK | 39.7 | 653.34(2) | <1 | 2 |
| | | | | | | | | VPTYVPLEMEKTYK | 39 | 566.63(3) | <1 | 2 |
| 46 | 5'-nucleotidase | XP_007427924.1 | *Python bivittatus* | 64 | 1 | 39.6 | 5% | VLLPSFLAAGGDGYH MLK | 39.6 | 630.34(3) | 2 | 2 |
| 47 | 5'-nucleotid | BAN89427.1 | *Ovophis okinaven* | 56 | 3 | 115.9 | 17% | HANFPILSANIRPK | 38.3 | 526.64(3,4) | <1 | 2 |
| | | | | | | | | YLGYLNVIFDDKGNVI | 38.9 | 657.69(3) | 1 | 2 |

| | | | | | | | | | | | |
|---|---|---|---|---|---|---|---|---|---|---|---|
| | ase, partial | | *sis* | | | | | K | | | | |
| | | | | | | | | GDSSNHSSG**N**LDISIV GDYIK | 38.7 | 727(3) | 8 | 2 |
| 48 | 5'-nucleotidase | V5NTD_GLOBB | | 6 | 1 | 39.8 | 28% | SFELTILHTNDVHAR | 39.8 | 438.98(3,4) | 1 | 2 |
| 49 | Snake venom 5'-nucleotidase-like, partial | XP_013921664.1 | *Thamnophis sirtalis* | 20 | 1 | 36.1 | 8% | VGSEKVGIIGYTTK | 36.1 | 484.61(3) | <1 | 2 |
| **Kunitz protease inhibitor (KPI)** | | | | | | | | | | | | |
| 50 | Kunitz protease inhibitor-II | ABD24041.1 | *Daboia russelii russelii* | 10 | 2 | 72.1 | 23% | FcFLRPDFGR | 37.4 | 657.82(2) | 2 | 10 |
| | | | | | | | | ENTNNFDTR | 34.7 | 555.74(2) | 3 | 10 |
| 51 | Protease inhibitor | AFB74191.1 | *Daboia russelii* | 10 | 3 | 107.2 | 43% | FcNLAPESGR | 39 | 575.77(2) | 1 | 9 |
| | | | | | | | | RIYYNPDSNKcEVFFY GGcGGNDNNFETR | 35.7 | 874.13(4) | 4 | 9 |
| | | | | | | | | IYYNPDSNKcEVFFYG GcGGNDNNFETR | 32.5 | 1113.13(3,4) | 2 | 9 |
| 52 | Kunitz protease inhibitor-IV | ABD24043.1 | *Daboia russelii russelii* | 9 | 1 | 32.6 | 33% | FYYNPASNQcQGFIYG GcGGNANNFETR | 32.6 | 1069.45(3) | <1 | 10 |
| 53 | Kunitz-type serine protease inhibitor 2 | VKT2_DABSI | | 7 | 1 | 38.6 | 25% | HDRPTFcNLAPESGR | 38.6 | 586.28(3,4) | <1 | 10 |
| **Hyaluronidase (Hyal)** | | | | | | | | | | | | |
| 54 | Hyaluronidase | ABI33937.1 | *Echis ocellatus* | 53 | 1 | 39.9 | 4% | HSDSNAFLHLFPESFR | 39.9 | 476.73(4) | 2 | 3 |

| | | | | | | | | | | | | |
|---|---|---|---|---|---|---|---|---|---|---|---|---|
| **Aminopeptidase (AP)** | | | | | | | | | | | | |
| 55 | xaa-Pro aminope ptidase 2 isoform X1 | XP_039 1 86984. 1 | *Crotalus tigris* | 77 | 5 | 192 | 9% | LSAYIVPNTDAHLSEY VAER | 41 | 750.02(3) | <1 | 1 |
| | | | | | | | | LADDFMGSTWQEK | 36.3 | 764.34(2) | <1 | 1 |
| | | | | | | | | VEDYDQIGASLR | 39.4 | 683.34(2) | <1 | 1 |
| | | | | | | | | VLMGNIDLSK | 40 | 545.30(2) | 1 | 1 |
| | | | | | | | | VVSLVPYAR | 35.3 | 502.30(2) | <1 | 1 |
| 56 | Xaa-Pro aminope ptidase 2 | JAG658 7 4.1 | *Boiga irregulari s* | 77 | 1 | 31.9 | 7% | LEDVALVVPAK | 31.9 | 577.35(2) | 7 | 1 |
| **Nerve Growth Factor (NGF)** | | | | | | | | | | | | |
| 57 | nerve growth factor, NGF | AAA032 8 2.1 | *Vipera russelli* | 13 | 3 | 114. 2 | 24% | HWNSYcTTTDTFVR | 34.8 | 596.60(3) | <1 | 6,8 |
| | | | | | | | | FIRINTAcVcVISR | 39.2 | 570.30(3) | <1 | 6,8 |
| | | | | | | | | INTAcVcVISR | 40.2 | 646.83(2) | <1 | 8 |
| **Carboxypeptidase (CP)** | | | | | | | | | | | | |
| 58 | Carboxy peptidas e E-like | AFJ496 0 9.1 | *Crotalus adamant eus* | 54 | 1 | 40.5 | 3% | AASQPGELKDWFVG R | 40.5 | 554.28(3) | 5 | 3 |
| **DNase** | | | | | | | | | | | | |
| 59 | Deoxyrib onucleas e-2- alpha, partial | ETE732 0 6.1 | *Ophioph agus hannah* | 46 | 1 | 39.7 | 3% | TLNQLYLEAANR | 39.7 | 703.38(2) | 2 | 3 |
| **Vascular Endothelial Growth Factor (VEGF)** | | | | | | | | | | | | |
| 60 | VR-1 precursor | ACN220 4 6.1 | *Daboia russelii russelii* | 16 | 1 | 75.1 | 10% | cSGccTDESMKcTPV G KHTADIQIMR | 35.3 | 609.27(5) | <1 | 9 |
| **Glutaminyl-peptide Cyclotransferases (GC)** | | | | | | | | | | | | |
| 61 | Glutamin yl- peptide | AFE847 6 2.1 | *Daboia russelii* | 42 | 6 | 223. 9 | 21% | MWQNDLHPIMIER | 39.4 | 561.61(3) | 2 | 4 |
| | | | | | | | | TFSNIISTLNPLAK | 37.3 | 759.93(2) | <1 | 4 |
| | | | | | | | | LIFFDGEEAFVR | 38.4 | 721.87(2) | 3 | 4 |

| | | | | | | | | | | | | |
|---|---|---|---|---|---|---|---|---|---|---|---|---|
| | cyclotran sferases | | | | | | | WSPSDSLYGSR | 36.6 | 627.79(2) | 1 | 4 |
| | | | | | | | | NPVFPVYFLNTAR | 38.8 | 769.41(2) | <1 | 4 |
| | | | | | | | | GVPILHLIPSPFPR | 33.4 | 514.98(3) | <1 | 4 |
