# Supplementary material for: Comparative Proteomics, Functional Characterization and Immunological Cross-Reactivity Studies on Russell’s Viper Venom from Two Distinct Geographical Regions in South India
Source: Int J Mol Sci. 2025 Oct 7;26(19):9734. doi: 10.3390/ijms26199734 (PMC12524627; doi:10.3390/ijms26199734)
Supplement: Supplementary file 1 [file ijms-26-09734-s001.zip › Table S3.pdf]

Supplementary Table S3: Spectrum reports of the proteins identified uniquely from RVwg.

3 (a). DNase: 3% coverage

Peptide sequence: TLNQLYLEAANR

ETE73206.1 (100%), 45,572.3 Da
Deoxyribonuclease-2-alpha, partial [Ophiophagus hannah]
1 exclusive unique peptides, 1 exclusive unique spectra, 1 total spectra, 12/403 amino acids (3% coverage)

```
M I F F F V G N C F   K S P V R T V P L R   W L R Y L S S T P S   G A S E K A S Y G Q   G F C R I L M L L I   L A L S L A L T Y D
V V Y G S I S C Y D   D S G E P V D W F I   V Y K L P K S H H S   S P V E G M R Y M Y   Q D G H T Q G W V K   G R S L M N S T E G
A V G R T L N Q L Y   L E A A N R Q R E K   E M A Y V L Y N D E   P P K E N F S G S K   G H T K G V I L L D   R T Q G F W L V H S
T P R F P P R L D E   E Y A W P H T A L T   Y G Q S F L C V T Y   P Y S Q F K A I G N   Q L L F T D P H V Y   D Y Q V Q G A F A R
D L P A L L N A S E   G E H V Q E E P W N   S Q V T L T S K G G   K E F V S F A K F S   F F D D D L Y S G W   L A Q A L A T D L Y
V E F W L K S S G V   L A S N C S G Q Y R   V Y N V E Q L A F Q   D P A T S F F S T S   D H S K W C V G I E   R D S G W A C V G D
M N R N H A E E H R   G G G T I C C H D V   T I W K S F H S L V   Q N Y T D C D T T G   Y G L
```

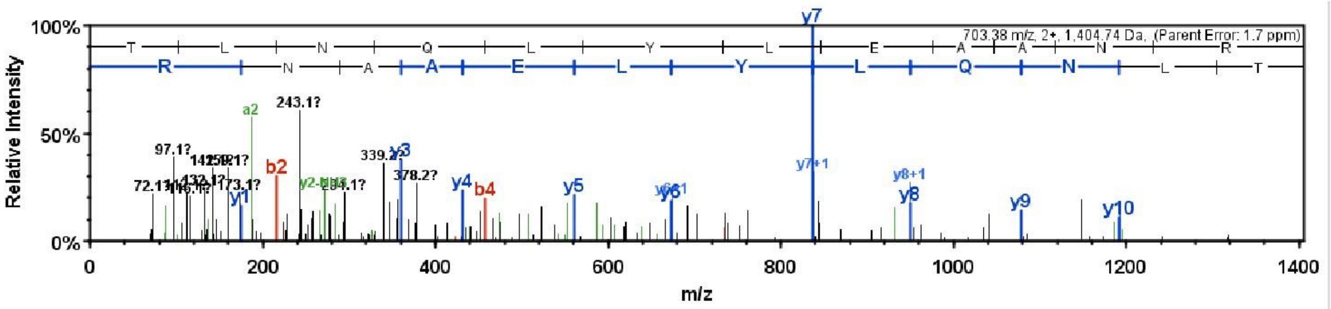

B	B Ions	B+2H	B-NH3	B-H2O	AA	Y Ions	Y+2H	Y-NH3	Y-H2O	Y
1	102.1			84.0	T	1,405.7	703.4	1,388.7	1,387.7	12
2	215.1			197.1	L	1,304.7	652.9	1,287.7	1,286.7	11
3	329.2		312.2	311.2	N	1,191.6	596.3	1,174.6	1,173.6	10
4	457.2		440.2	439.2	Q	1,077.6	539.3	1,060.5	1,059.6	9
5	570.3		553.3	552.3	L	949.5	475.3	932.5	931.5	8
6	733.4	367.2	716.4	715.4	Y	836.4	418.7	819.4	818.4	7
7	846.5	423.7	829.4	828.5	L	673.4	337.2	656.3	655.4	6
8	975.5	488.3	958.5	957.5	E	560.3		543.3	542.3	5
9	1,046.6	523.8	1,029.5	1,028.5	A	431.2		414.2		4
10	1,117.6	559.3	1,100.6	1,099.6	A	360.2		343.2		3
11	1,231.6	616.3	1,214.6	1,213.6	N	289.2		272.1		2
12	1,405.7	703.4	1,388.7	1,387.7	R	175.1		158.1		1

3 (b). Hyaluronidase: 4% coverage

Peptide sequence: HSDSNAFLHLFPESFR

ABI33937.1 (99%), 52,545.3 Da
hyaluronidase [Echis ocellatus]
1 exclusive unique peptides, 1 exclusive unique spectra, 1 total spectra, 16/449 amino acids (4% coverage)

MYHLWIKCLA AWIFLKRFNG VHVMHAKAPM YPNEPFLVFW NAPTTQCRLR YKVDLDLKTF
HIVANANDTL SGSAVTIFYP THLGIYPHID DRGHFFHGII PQNESLTKHL DKSKSDINRI
IPLKTFHGLG VIDWENWRPQ WDRNWGNKNV YRNRSIQFAR DLHPELSENK IRRLAKAEYE
KAAKSFMRDT LLLAEEMRPD GYWGYYLYPD CQNYDYKTKG DQYTGKCPDI EMSRNDQLLW
LWRESTALFP NVYLEIILRS SDNALKFVHH RLKESMRIAS MAREDYALPV FVYARPFYAY
TFEPLTQEDL VTTVGETAAM GAAGIVFWGS MQYASTVDSC QKVKKYMNGP LGRYIINVTT
AAKICSHALC RKNGRCVRK**H** **SDSNAFLHLF** **PESFR**IMVHA NATEKKVIVK GKLELENLIY
LRENFMCQCY QGWQGLYCEE YSIKDIRKI

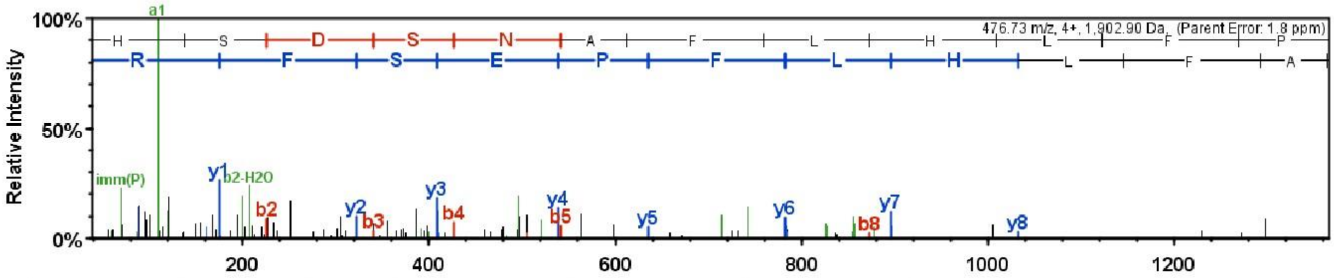

B	B Ions	B+2H	B-NH3	B-H2O	AA	Y Ions	Y+2H	Y-NH3	Y-H2O	Y
1	138.1	69.5			H	1,903.9	952.5	1,886.9	1,885.9	16
2	225.1	113.1		207.1	S	1,766.8	883.9	1,749.8	1,748.8	15
3	340.1	170.6		322.1	D	1,679.8	840.4	1,662.8	1,661.8	14
4	427.2	214.1		409.1	S	1,564.8	782.9	1,547.8	1,546.8	13
5	541.2	271.1	524.2	523.2	N	1,477.8	739.4	1,460.7	1,459.7	12
6	612.2	306.6	595.2	594.2	A	1,363.7	682.4	1,346.7	1,345.7	11
7	759.3	380.2	742.3	741.3	F	1,292.7	646.8	1,275.7	1,274.7	10
8	872.4	436.7	855.4	854.4	L	1,145.6	573.3	1,128.6	1,127.6	9
9	1,009.4	505.2	992.4	991.4	H	1,032.5	516.8	1,015.5	1,014.5	8
10	1,122.5	561.8	1,105.5	1,104.5	L	895.5	448.2	878.4	877.5	7
11	1,269.6	635.3	1,252.6	1,251.6	F	782.4	391.7	765.4	764.4	6
12	1,366.7	683.8	1,349.6	1,348.6	P	635.3	318.2	618.3	617.3	5
13	1,495.7	748.4	1,478.7	1,477.7	E	538.3	269.6	521.2	520.3	4
14	1,582.7	791.9	1,565.7	1,564.7	S	409.2	205.1	392.2	391.2	3
15	1,729.8	865.4	1,712.8	1,711.8	F	322.2	161.6	305.2		2
16	1,903.9	952.5	1,886.9	1,885.9	R	175.1	88.1	158.1		1

3 (c). Venom endothelial growth factors (VEGF): 10% coverage

Peptide sequence: cTPVGKHTADIQIMR

ACN22046.1 (100%), 16,277.5 Da
VR-1 precursor [Daboia russelii russelii]
1 exclusive unique peptides, 2 exclusive unique spectra, 4 total spectra, 15/144 amino acids (10% coverage)

M A A Y L L A V A I   L F C I Q G W P S G   T V Q G Q V R P F L   D V Y E R S A C Q T   R E T L V S I L Q E   H P D E I S D I F R
P S C V A V L R C S   G C C T D E S M K **C**   **T P V G K H T A D I**   **Q I M R** M N P R T H   S S K M E V M K F M   E H T A C E C R P R
W K Q G E P E G P K   E P R R G G V R A K   F P F D

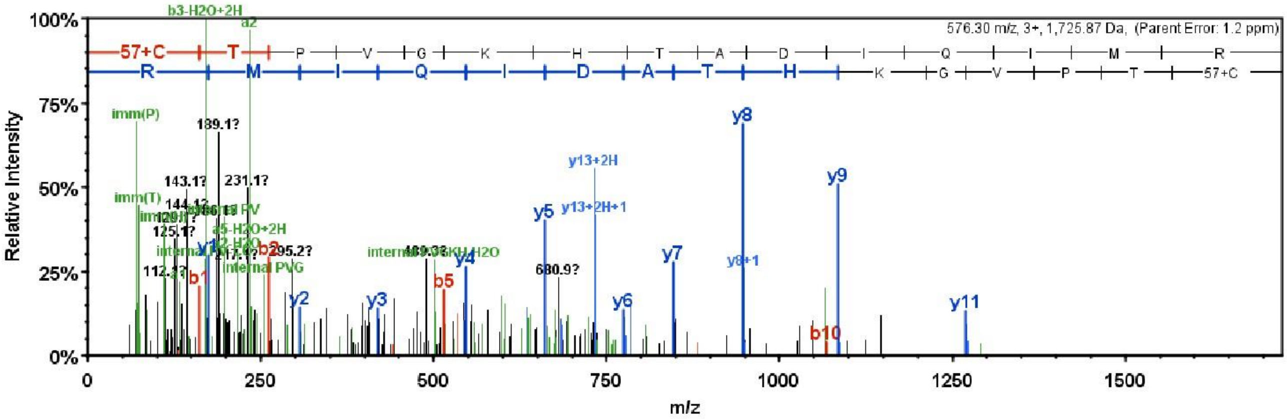

B	B Ions	B+2H	B-NH3	B-H2O	AA	Y Ions	Y+2H	Y-NH3	Y-H2O	Y
1	161.0	81.0			C+57	1,726.9	863.9	1,709.8	1,708.9	15
2	262.1	131.5		244.1	T	1,566.8	783.9	1,549.8	1,548.8	14
3	359.1	180.1		341.1	P	1,465.8	733.4	1,448.8	1,447.8	13
4	458.2	229.6		440.2	V	1,368.7	684.9	1,351.7	1,350.7	12
5	515.2	258.1		497.2	G	1,269.7	635.3	1,252.6	1,251.7	11
6	643.3	322.2	626.3	625.3	K	1,212.7	606.8	1,195.6	1,194.6	10
7	780.4	390.7	763.4	762.4	H	1,084.6	542.8	1,067.5	1,066.5	9
8	881.4	441.2	864.4	863.4	T	947.5	474.3	930.5	929.5	8
9	952.5	476.7	935.4	934.5	A	846.5	423.7	829.4	828.4	7
10	1,067.5	534.3	1,050.5	1,049.5	D	775.4	388.2	758.4	757.4	6
11	1,180.6	590.8	1,163.6	1,162.6	I	660.4	330.7	643.4		5
12	1,308.6	654.8	1,291.6	1,290.6	Q	547.3	274.2	530.3		4
13	1,421.7	711.4	1,404.7	1,403.7	I	419.2	210.1	402.2		3
14	1,552.8	776.9	1,535.7	1,534.8	M	306.2	153.6	289.1		2
15	1,726.9	863.9	1,709.8	1,708.9	R	175.1	88.1	158.1		1
